# Optogenetic perturbations reveal the dynamics of an oculomotor integrator

**DOI:** 10.3389/fncir.2014.00010

**Published:** 2014-02-28

**Authors:** Pedro J. Gonçalves, Aristides B. Arrenberg, Bastian Hablitzel, Herwig Baier, Christian K. Machens

**Affiliations:** ^1^Group for Neural Theory, Departement d'Etudes Cognitives, INSERM U960, École Normale SupérieureParis, France; ^2^Champalimaud Neuroscience Program, Centro Champalimaud – Champalimaud Centre for the UnknownLisbon, Portugal; ^3^Gatsby Computational Neuroscience Unit, University College LondonLondon, UK; ^4^Neuroscience Program, Department of Physiology, University of California San FranciscoSan Francisco, CA, USA; ^5^Faculty of Biology, Center for Biological Signaling Studies, University of FreiburgFreiburg, Germany; ^6^Max Planck Institute of NeurobiologyMartinsried, Germany

**Keywords:** neural integrator, optogenetics, model, zebrafish, oculomotor system, network dynamics

## Abstract

Many neural systems can store short-term information in persistently firing neurons. Such persistent activity is believed to be maintained by recurrent feedback among neurons. This hypothesis has been fleshed out in detail for the oculomotor integrator (OI) for which the so-called “line attractor” network model can explain a large set of observations. Here we show that there is a plethora of such models, distinguished by the relative strength of recurrent excitation and inhibition. In each model, the firing rates of the neurons relax toward the persistent activity states. The dynamics of relaxation can be quite different, however, and depend on the levels of recurrent excitation and inhibition. To identify the correct model, we directly measure these relaxation dynamics by performing optogenetic perturbations in the OI of zebrafish expressing halorhodopsin or channelrhodopsin. We show that instantaneous, inhibitory stimulations of the OI lead to persistent, centripetal eye position changes ipsilateral to the stimulation. Excitatory stimulations similarly cause centripetal eye position changes, yet only contralateral to the stimulation. These results show that the dynamics of the OI are organized around a central attractor state—the null position of the eyes—which stabilizes the system against random perturbations. Our results pose new constraints on the circuit connectivity of the system and provide new insights into the mechanisms underlying persistent activity.

## Introduction

Neural activity deep within the nervous system or close to the motor periphery is largely driven by a combination of intrinsic neuronal properties and recurrent feedback among neurons. Such activity is almost always dynamic, changing either fast, as in central pattern or sequence generators (Marder and Bucher, 2001; Hahnloser et al., 2002), or slowly, as in the neural integrators that have been found at many levels of the nervous system (Robinson, 1968; Pastor et al., 1994; Gold and Shadlen, 2001; Wong et al., 2007; Goldman et al., 2009). A key question in neuroscience is how neural systems generate and control these internal dynamics through links between individual neurons.

One of the simplest and best-studied systems to address this question is the oculomotor integrator (OI) for horizontal eye movements. Neurons in the OI are persistently active with a discharge rate that is directly proportional to the horizontal eye position (Lopez-Barneo et al., 1982; Delgado-García et al., 1989; Fukushima et al., 1992; McFarland and Fuchs, 1992; Aksay et al., 2000). This graded persistent activity typifies a simple form of short-term memory and shares many similarities with the persistent activity found in higher-order brain areas during working memory (Brody et al., 2003; Major and Tank, 2004; Goldman et al., 2009). Several theoretical studies have shown how the persistent activity in the OI can be generated through precise recurrent synaptic feedback among neurons (Cannon et al., 1983; Cannon and Robinson, 1985; Seung, 1996; Seung et al., 2000; Aksay et al., 2007). This body of work has led to a network model of the OI that can essentially reproduce all experimentally measured features in the goldfish, such as the distribution of tuning curves (Seung et al., 2000; Aksay et al., 2007), the correlations between simultaneously recorded neurons, the generation of saccades, or the system's response to unilateral silencing (Aksay et al., 2007). Moreover, several candidate mechanisms were pointed out to explain the remarkable robustness of the system (Koulakov et al., 2002; Goldman et al., 2003; Moreau and Sontag, 2003).

Here, we show that this network model can be understood as a particular instantiation of a class of models, all of which can explain the shared experimental features across animals. The models are only distinguished by the specific ratio of excitatory and inhibitory inputs to the neurons. Each network model fully specifies the dynamics of the OI. While the dynamics are similar in the system's normal operating regime, they are distinct outside of this regime. Specifically, different network models relax differently toward the persistent activity states. Consequently, different instantiations of the network models make different predictions on how the OI will react to perturbations. These predictions can be tested with the recent advances of optogenetic tools which allow us to manipulate systems with high spatio-temporal precision (Nagel et al., 2003; Boyden et al., 2005; Lima and Miesenböck, 2005; Han and Boyden, 2007; Zhang et al., 2007a,b; Douglass et al., 2008; Huber et al., 2008; Arrenberg et al., 2009; Schoonheim et al., 2010; Fenno et al., 2011).

In systems that are strongly driven by their own internal dynamics, the outcome of a perturbation depends on a combination of the externally applied stimulation and the intrinsic network dynamics. In many instances, neural systems are not sufficiently well known to disentangle the two and make precise and quantitative predictions. The modeling approach we pursue here, however, provides the opportunity to predict the influence of these two competing effects, and by comparison with experimental data, adjust the model, and further our understanding of the system.

Here, we test these model predictions using transgenic zebrafish expressing either halorhodopsin (NpHR), a light-driven chloride pump, or channelrhodospin (ChR2), a light-activated cation channel (Zhang et al., 2007a,b; Arrenberg et al., 2009; Fenno et al., 2011). We show that these instantaneous and small perturbations of the OI network yield crucial insights into the dynamics around the system's normal operating regime. We show that only one of the network models can explain all the data. This model suggests a dominance of unilateral self-excitation, concurrent with a weak coupling between OI cells in the left and right hemisphere. While the stable states still form a line attractor in our new model, the dynamics around the line attractor differ from those of previously proposed models. Specifically, the dynamics are organized around the center of the line attractor which corresponds to the null position of the eyes. This network arrangement could be preferable for the animal, since any perturbations due to noise or synaptic mistuning will cause drifts toward the resting state, instead of drifting toward extreme population activities and eye positions.

## Results

### Network models of the oculomotor integrator

**Figure 1:**
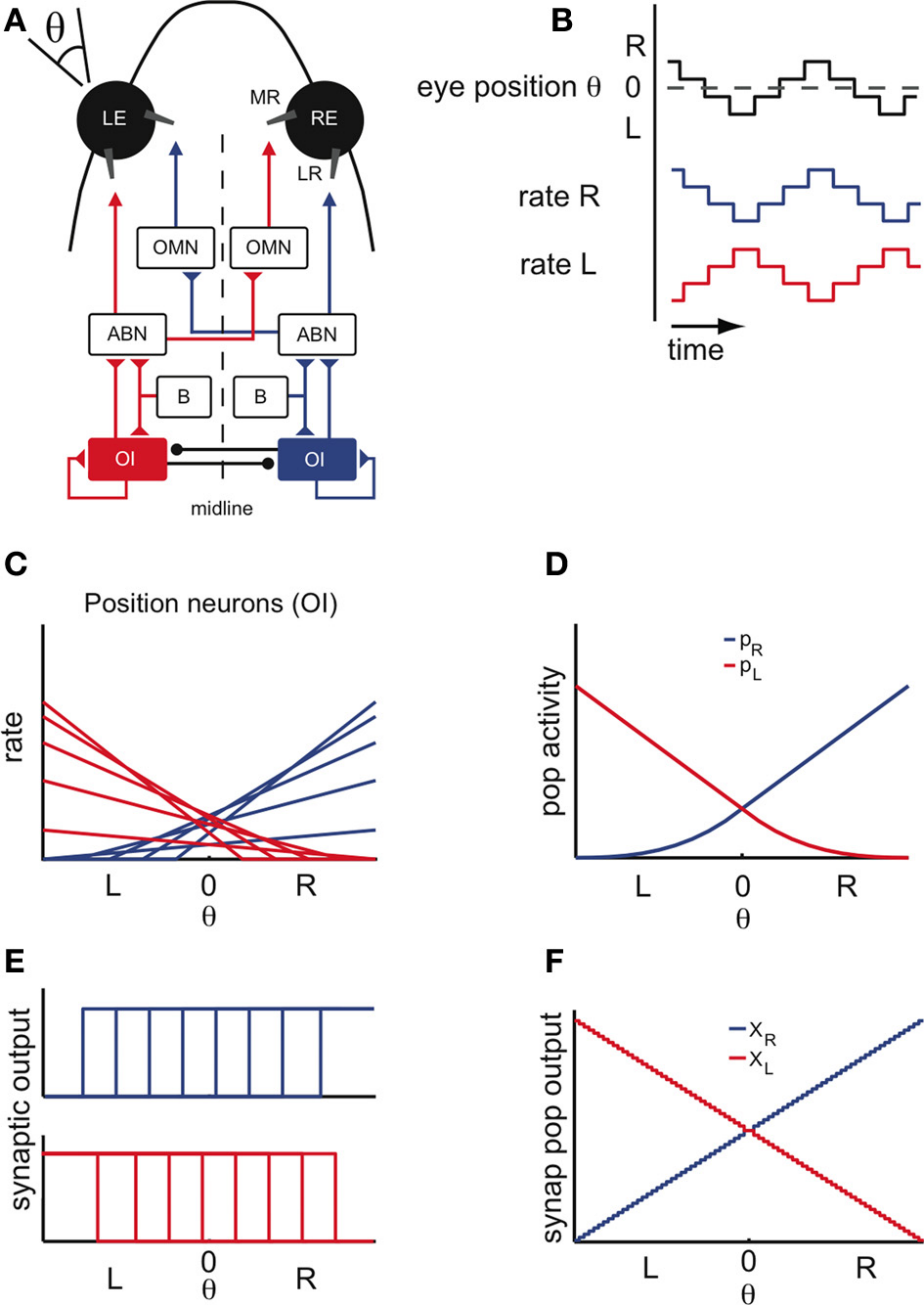
**Properties of the oculomotor integrator (OI). (A)** Schematic of the oculomotor system for horizontal eye movements. The neurons carrying a position signal are located in the oculomotor integrator (OI) and are thought to integrate (in the sense of calculus) the velocity signal from the burst nucleus (B). Studies of pairwise neural correlations suggest recurrent excitatory connections within each side of the OI and mutual-inhibition between opposite sides. The burst nucleus (B) generates the saccadic eye movements. Position and velocity signals are provided to the muscles (LR: lateral rectus; MR: medial rectus) via excitation of the motoneurons in the abducens nucleus (ABN) and the contralateral oculomotor nucleus (OMN). **(B)** Idealized eye position trace and concurrent position cell activity. In the absence of external visual feedback, the fish moves its eyes spontaneously in scanning cycles, side to side (left side L; right side R; central position 0), alternating stable fixations with saccades. Stable firing rates of the position cells are linearly related to eye position during behavior. **(C)** Tuning curves of right (blue) and left (red) position cells. **(D)** Overall population activity of the left (*p*_*L*_) and right side (*p*_*R*_), defined as the sum over the activity of all individual cells. **(E)** Single-cell synaptic tuning curves for right (top) and left (bottom) position cells, with thresholds assumed to span the whole motor range. **(F)** Synaptic population activities of right (*X*_*R*_) and left (*X*_*L*_) half of the integrator as a function of eye position. Note the staircase shape of the curves, a consequence of the saturation of the single-cell synaptic activity curves.

The main anatomical and physiological features of the OI are summarized in Figures 1A–C. The OI is located in the hindbrain and composed of two bilaterally symmetric neuronal nuclei (Figure 1A). In goldfish, these nuclei consist of around *N* = 40 neurons (Pastor et al., 1994; Aksay et al., 2000), and are referred to as Area I. Quick horizontal eye movements (saccades) are generated by motoneurons in the abducens and oculomotor cranial nuclei (ABN, OMN, Figure 1A), which receive a velocity command from excitatory burst cells. The OI receives a collateral of the velocity signal and integrates it in the mathematical sense to create a position signal. The firing rates of the OI neurons are therefore linearly related to eye position (Figures 1B,C). The slope of their tuning curves is higher for neurons with more central eye position firing thresholds and lower for neurons with contraversive, peripheral eye position thresholds (Aksay et al., 2000), a property that has been called “recruitment order” (see Materials and Methods). Neurons from the same side are usually positively correlated, whereas neurons from opposite sides are negatively correlated, suggesting that the two sides are coupled by mutual inhibition and self-excitation (Aksay et al., 2003) as shown in Figure 1A. The firing rates of these “position” neurons remain stable in the absence of visual feedback and provide the signal that controls fixation of the horizontal eye position (Mensh et al., 2004).

These observations indicate that the network of position neurons can maintain a continuum of persistent firing rates over several seconds, i.e., the time scale of a typical eye fixation. Since only these persistent firing patterns are observed, the population activity of the two sides must be highly constrained. We use these constraints to reduce the network dynamics to the dynamics of the two interacting populations, which we describe by their summed activity (Figures 1D,F – see also **Figures A1D–F**). We will write *X*_*L*_ and *X*_*R*_ for the left- and right-side population output, measured as the resulting post-synaptic conductances (Figures 1E,F) and will refer to this population output as “population activity.”

As further discussed below, our modeling framework requires that the synaptic currents saturate to balance the progressive recruitment of neurons. Although such synaptic non-linearities are yet to be found in the OI, we here assume their presence. As discussed in the Materials and Methods, we choose step input-output functions to simplify the model tuning but can relax that assumption by using smoother sigmoidal functions (see Appendix). Given this choice, the synaptic output tuning curves are idealized, saturated versions of the firing rate tuning curves (Figures 1C,E). For simplicity of the model tuning, we also assume that the single-cell synaptic outputs have thresholds spanning the whole eye position range. This assumption can be reconciled with the data if the synaptic currents have different thresholds (Aksay et al., 2007). In consequence, the left- and right-side synaptic population outputs *X*_*L*_ and *X*_*R*_ resemble a staircase function where each step is caused by the synaptic input-output function of a single neuron (Figures 1E,F—see also Materials and Methods). Since there are many neurons involved, these population outputs approximate linear functions of the eye position.

**Figure 2:**
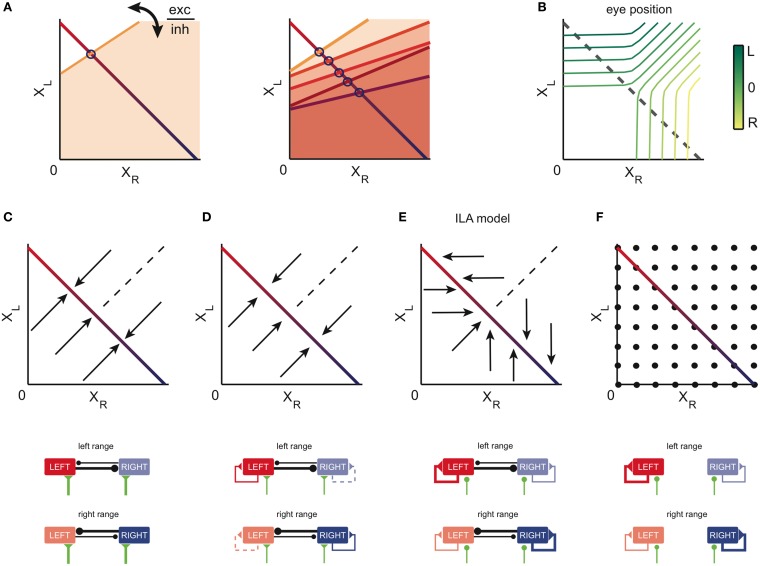
**Network models of the OI: construction and dynamics. (A)** State space of the network model. We assume that the state is uniquely described by the value of the left and right population activities, *X*_*L*_ and *X*_*R*_. The red-blue colored line corresponds to the stable equilibrium points (line attractor) of the system. Left eye positions are on the red part of the line, right eye positions on its blue part. Blue circles indicate the eye position thresholds of the tuning curves of neurons from the right population along the line attractor. Orange lines correspond to the thresholds of firing for other states of the system (orange shaded areas indicate the corresponding regions above the threshold). Left: the respective slopes are given by the relative strength of the (self-) excitatory and (cross-) inhibitory input into each neuron. Right: as the eyes move from left to right, more and more neurons from the right population get recruited (compare Figure 1C). **(B)** Mapping of position population activities onto eye position. The colored iso-lines correspond to different eye positions θ in the (*X*_*R*_, *X*_*L*_) space. The gray dashed line indicates the line attractor and thereby the stable equilibrium states of the system. **(C–F)** Example models generated within our theoretical framework. Top: state space of the example models. Arrows indicate the direction of the dynamics. Points indicate stationary states. Bottom: connectivity. Thick, thin, and dashed connectivity lines correspond to strong, weak, and very weak neural connections. The thickness of these connections corresponds to the absolute of the sum of the excitatory post-synaptic potentials (EPSP) and inhibitory post-synaptic potentials (IPSP) of the neurons post-synaptic to the connection. The green lines depict external (e.g., vestibular) inputs to the integrator areas. **(C)** A model in which neural activity is kept persistent due to mutual inhibition between the sides. **(D)** A model in which both mutual inhibition and self-excitation provide the stability of the persistent states. **(E)** The ILA model, which can reproduce the goldfish inactivation results (Aksay et al., 2007). Here, the dynamics are mostly unidirectional and involve only one population on each side (arrows are horizontal or vertical). In the left motor range (bottom panel, top), the left network sustains its firing through self-excitation, whereas the right network is passive, given the weakness of the recurrent inputs. Therefore, the dynamics are dominated by the left network. In the right range (bottom panel, bottom), the inputs and dynamics are reversed. **(F)** A model in which persistent activity is generated through self-excitation only. Here, both sides are completely independent, and every combination of population activities is stable.

The essence of our network models is shown in Figure 2 (see also Materials and Methods; a Matlab-based implementation of the models is available in the online supplementary information). In Figure 2A, we plot the population activities of Figure 1F against each other. Given the staircase shape of the population outputs, the resulting relationship is composed of multiple points on a line, which we here approximate by the red-blue line. This line illustrates the persistent firing states of the system, which range from virtual silence on the right and strong activity on the left side (*X*_*R*_ = 0, *X*_*L*_ large, red color) to virtual silence on the left and strong activity on the right (*X*_*R*_ large, *X*_*L*_ = 0, blue color). Different eye positions correspond to different points on this line (Figure 2B; see also below), and each point corresponds to a stable mode of firing for the network. The line is therefore often called a “line attractor” (Seung, 1996).

In the absence of synaptic input, neurons cease to fire given the neuronal leak. Therefore, for neural activities to be stable in a network in the absence of an external input, the recurrent network input to each neuron has to exactly match the neuronal leak (see Materials and Methods). An imbalance between those would cause either runaway excitation or inhibition. As the eye position moves from left to right, this balance of inputs and neuronal leaks needs to be maintained despite the non-linear distortions (such as thresholds or saturations) introduced by the biophysics of neurons and synapses. The resulting distortions can be compensated by the successive recruitment of neurons from one side and the suppression of neurons from the opposite side, as prescribed by the recruitment order (Figure 1C, see Materials and Methods, for details) (Seung, 1996; Seung et al., 2000; Aksay et al., 2007). It is important to note however that to obtain a line attractor in our network models, fine-tuning of the parameters is necessary, since changes in the parameters as small as 1% disrupt the line attractor dynamics.

Given the connectivity of the two sides of the system, the response of a neuron (on the right) is modeled as *x*_*R*_ = *H*(*aX*_*R*_ − *cX*_*L*_ + *h*), where parameter “*a*” determines the weight of the excitatory input from the right population *X*_*R*_, parameter “*c*” determines the weight of the inhibitory input from the left population *X*_*L*_, and parameter “*h*” models constant external inputs to the network. The function *H*(.) models the neuron's input-output function, and is either a threshold-linear function (in the case of a firing rate response) or a Heaviside function (in the case of a synaptic output response). The threshold of this input–output function corresponds to the set of points for which *aX*_*R*_ − *cX*_*L*_ + *h* = 0 (orange threshold line, Figure 2A). Any (*X*_*R*_, *X*_*L*_) combination that is below the orange line will make the right side neuron shown in Figure 2A fire.

The recruitment order fixes the thresholds of the neurons on the red-blue line as illustrated by the blue circles for a few example neurons (Figure 2A). However, the data do not specify how a neuron would respond to population activities *X*_*R*_ and *X*_*L*_ outside of this line, leaving a degree of freedom that is related to the relative strength of the self-excitatory input “*a*” and cross-inhibitory input “*c*” into each neuron (Figure 2A). Depending on how this threshold line is chosen for each neuron, the dynamics of the population activities outside of the line attractor change accordingly.

The dynamics of four exemplary models are illustrated in Figures 2C–F. Here, the arrows point in the direction in which the population activities evolve from different starting points. In Figure 2C, the line attractor is generated through mutual inhibition of the two sides. This configuration corresponds to one of the oldest models proposed for the OI (Cannon et al., 1983). In Figure 2D, the external excitatory connections are weakened, yet the resulting detrimental effect is compensated by weak self-excitation. This model was tested by Aksay et al. (2007), and is an extrapolation from the model in Seung et al. (2000): the authors here included mutual inhibition, while keeping orthogonal relaxation dynamics to the line attractor. We note that for both the mutual inhibition model and the weak self-excitation model, the dynamics outside of the attractor are orthogonal to the line, although the mutual inhibition model suggests faster dynamics (as indicated by the longer arrows).

The population dynamics of the model in Figure 2E were introduced by Aksay et al. (2007) to account for unilateral inactivation experiments in the goldfish. To obtain such dynamics in our modeling framework, the inhibitory and excitatory connections are set up so that each half of the oculomotor range is stabilized by an independent line attractor (ILA model). As a consequence, the population with the high activity (e.g., *X_R_*) does not change its activity when the other side's population activity (*X*_*L*_) is reduced. This situation is given when the left half of the system is silenced, which is equivalent to setting *X*_*L*_ = 0. Although the dynamics above the line attractor are unconstrained by experimental data, this model proposes that the dynamics above and below the line attractor are antiparallel to each other. We note that the ILA model captures the same population state space dynamics as the model suggested in Aksay et al. (2007), although the detailed implementation differs from the one in Aksay et al. (2007): the model does not incorporate input-output functions with high synaptic thresholds and uses a different distribution of tuning curves and cross-inhibition (see Materials and Methods).

A last example model is shown in Figure 2F. Here, the line attractor is stabilized through self-excitation only, and the inhibitory connections are non-existent. We note that this model is an extension of the model in Seung et al. (2000) from one population to two populations of excitatory neurons. In this case, any point outside of the line will be a potential stable fixed point as well. The system may still be confined to the line in practice, if the burst input during saccades always moves the population activities back onto the line.

### Unilateral instantaneous perturbations: model predictions

The network models allow us to predict precisely how a perturbation would affect the system. Most importantly, these perturbations can be observed at the level of the eye position which makes the predictions experimentally accessible. To link the population activities to the eye position, we note that the iso-eye-position curves are likely passing through a non-linear transform introduced in the abducens nucleus (Figure 2B; discrepancy of position cell and motoneuron tuning curves—for details see **Figure A3** and Materials and Methods). This bending of the curves also provides a simple explanation for the results of unilateral silencing of the OI in which the stabilization of eye position remains functional in only half the motor range and for roughly half the range of population activities (Aksay et al., 2007).

**Figure 3:**
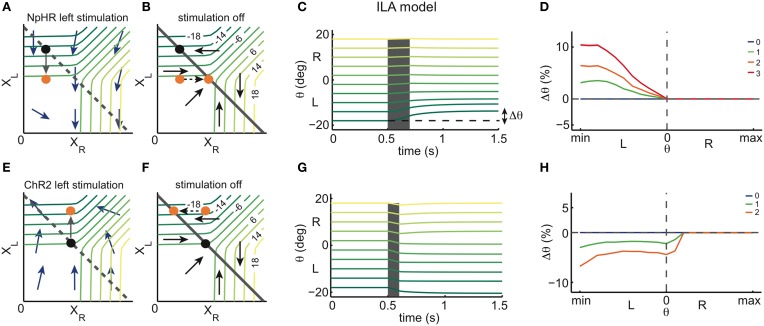
**Instantaneous perturbations of the left OI: simulations. (A)** State space of the ILA model during left inhibitory stimulation. The blue arrows correspond to the dynamics of the model during a stimulation and show that the left population activity is driven toward lower values, while the right population activity is largely unaffected. The black dot represents one example eye position and population activity (*X*_*R*_, *X*_*L*_) before stimulation. The orange dot represents the population activity and eye position after stimulation. The gray arrow corresponds to the movement from the initial to the final position. We note that the arrows are enlarged to clarify the direction of the movement, and do not correspond to the magnitude of the movements observed in simulations in **(C)**. The gray dashed line corresponds to the line attractor (which is overridden by the stimulation). **(B)** State space of the ILA network model after stimulation offset. Population activities relax back to the line attractor according to the dynamics of the intact system, given by the arrows. The colored iso-lines are annotated with the corresponding eye positions θ. The gray line corresponds to the line attractor. **(C)** Simulated eye position traces during left stimulation (gray shaded period) for the ILA model. Colors indicate the initial eye position (before the stimulation). The deflections of the eye position depend on the initial eye position. Only initial eye positions in the left half of the motor range can be affected by the stimulation. **(D)** ILA model predictions for the eye position changes, Δθ, as a function of the initial eye position, θ. Color indicates the intensity of stimulation. Positive Δθ correspond to changes toward the right, and negative Δθ correspond to changes toward the left. Eye position changes are measured as a proportion of the whole motor range, and “min” and “max” are the minimum and maximum eye positions of the motor range. For each intensity of stimulation, the respective plot corresponds to averages over 4000 stimulations in each of 21 eye positions. **(E)** State space of the ILA model during left excitatory stimulation. As can be seen by the dynamics, the left population activity increases, while the right population activity is largely unaffected. **(F)** Relaxation to the line attractor after stimulation. **(G)** Simulated eye position traces during left stimulation (gray shaded period). Same format as panel **(C)**. Only initial eye positions in the left half of the motor range can be affected by the stimulation. **(H)** ILA model predictions for the eye position changes, Δθ, as a function of the initial eye position, θ. Simulations were performed as in panel **(D)**.

We can then simulate the response of the models to unilateral instantaneous inhibition and excitation, mimicking optogenetic stimulations with NpHR and ChR2, respectively. This idea is illustrated in Figure 3, where we focus on one of the network models, the ILA model (Figure 2E).

Figures 3A–D shows the effect of inhibiting the left half of the OI in the ILA model. Due to the extra inhibition, the left population activity *X*_*L*_ decreases immediately, as indicated by the arrows in Figure 3A. If the eye position before stimulation (initial eye position) is in the left range (black point), the inhibition causes the system state to cross θ isolines transversely so that the eye makes large movements to the right (orange point). If the initial eye position is in the right range, the system state shifts mostly in parallel to θ isolines, and the eye movements are small or null. After switching-off the inhibition stimulus, the system relaxes back to the line according to the dynamics of the intact system (Figure 3B). We note that if the initial eye position is in the left range, *X*_*R*_ increases, thereby moving the eye further to the midline. If the initial position is in the right range, *X*_*L*_ increases and *X*_*R*_ does not change after the stimulation is turned-off, so that the system returns to its initial state and the net eye movement is null (Figures 3A,B). We can extend this perturbation analysis to all initial eye positions, i.e., all points on the line attractor. Naturally, the results will depend on both the length and intensity of the stimulation. We used a brief stimulation (200 ms) and varying stimulation intensities. The net eye movement resulting from the combination of stimulation and relaxation was measured as the difference Δθ in eye positions just before the stimulation and 1 s after the stimulation (see simulations in Figure 3C). Large Δθ are observed when the initial eye position is in the left range, and negligible Δθ when the initial position is in the right range, (Figure 3D).

With similar reasoning, we can explore the system's response to excitatory perturbations (Figures 3E–H). During left excitation of the ILA model (Figure 3E), the left population activity, *X*_*L*_, increases. After the stimulation, the system state relaxes back to the line attractor with the dynamics of the intact system (Figure 3F). If the initial eye position is in the left range, the value of *X*_*L*_ stays constant, and the right population activity, *X*_*R*_, decreases (Figures 3F,G). Altogether, the eye makes therefore large movements to the left. If the initial position is in the right range, the system state moves mostly in parallel to θ isolines and the eye movements are small or null. Consequently, the perturbations Δθ of eye position occur mostly on the ipsilateral side to the stimulation, similar to the inhibitory perturbations, but with opposite sign (Figures 3G,H).

In both cases, the perturbations Δθ reflect the relaxation dynamics of the system, i.e., the dynamics of the intact system. By measuring these simulated perturbations for the full range of initial eye positions, we can cover all points of the line attractor. While we have illustrated these perturbations for the ILA model, we can perform similar predictions for the whole range of models. Conversely, we can measure the system's response to perturbations in optogenetic experiments, and then simply infer the dynamics of the system around the line attractor that are consistent with the experiments.

We note that we here modeled NpHR stimulations as divisive and ChR2 stimulations as additive. This distinction is based on electrophysiological recordings from the caudal zebrafish hindbrain (not limited to OI cells) which showed that NpHR stimulations induce a change in firing rate that is dependent on the initial firing rate, while for ChR2 stimulations no such effect was observed in the (small) range of firing rates tested (**Figure A9**). Therefore, for simplicity, we modeled the effect of ChR2 stimulation as being additive. However, assuming a subtractive influence of NpHR on population activity, or a multiplicative influence of ChR2 yields qualitatively the same results (Gonçalves, 2012) (simulation data not shown), and does not impede our ability to infer the overall dynamics from measurements.

### Unilateral optogenetic perturbations: results of NpHR experiments

**Figure 4:**
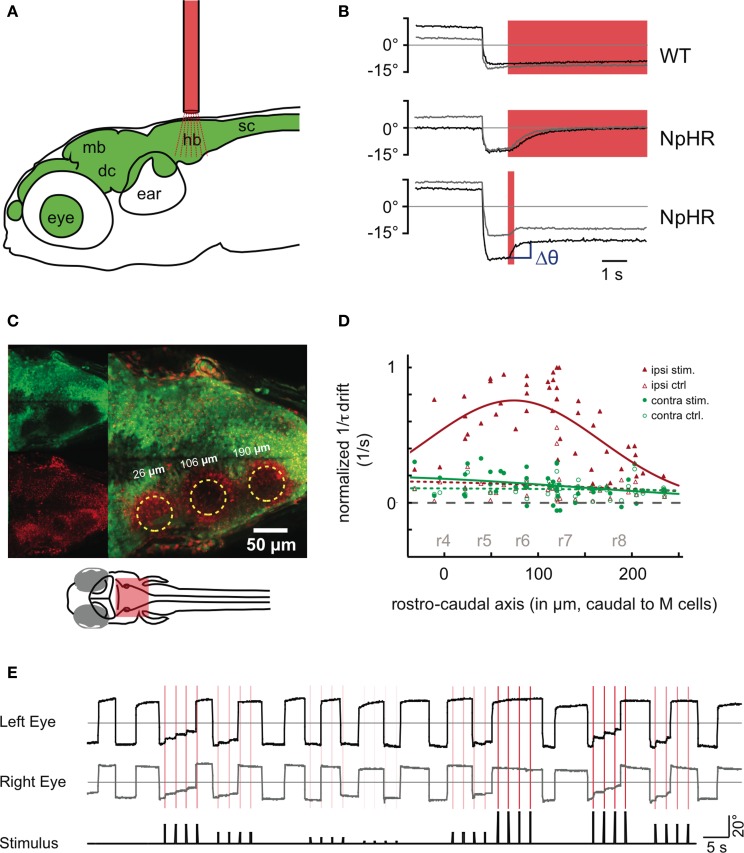
**Halorhodopsin (NpHR) stimulation in the hindbrain induces drifts in the eye position. (A)** An optic fiber (red) is placed above the zebrafish head (6 dpf) to perturb neuronal activity in the brain (green). hb, hidbrain; sc, spinal cord; mb, midbrain; dc, diencephalon. **(B)** Bilateral NpHR stimulation in the hindbrain induces eye drifts toward the null position. Saccades to the left side are shown for both the left eye (black trace) and the right eye (gray trace). The red shade indicates the stimulation period (6 s or 200 ms). Top: Wildtype animal. Middle: NpHR expressing animal, 6 s stimulation. Bottom: NpHR expressor, 200 ms stimulation. Δθ (blue) is the change in eye position from before the stimulation to 1 s after the stimulation. **(C,D)** An optic fiber (50 μm diameter) was placed at different rostro-caudal positions of the hindbrain and the resulting eye drift magnitudes during unilateral stimulation were measured. **(C)** A 5 dpf hindbrain transgenic for *Et(E1b:Gal4-VP16)s1101t, Tg(UAS:NpHR-mCherry)s1989t*, and *Tg(UAS:Kaede)s1999t*. The green channel contains non-converted Kaede signal (upper left), while the red channel contains converted Kaede and mCherry fluorescence. Kaede was photoconverted from green to red at three fiber positions (yellow circles). A maximum projection of selected optical slices is shown. Note: The broadly distributed red speckles are NpHR-mCherry fluorescent protein aggregates. **(D)** Inverse of the time constants of drift τ (in (1/s), see Materials and Methods—Data Analysis) induced by NpHR stimulations at different rostro-caudal levels, relative to the Mauthner cell position (*n* = 7 animals). The data is split according to the eye position before stimulation. Eye positions (averaged across the two eyes) on the same side as the stimulation (left) are plotted in red (ipsiversive) and eye positions on the opposite side (contraversive) are plotted in green. Control trials (open symbols, no light stimulation) are included. The data points are fitted by Gaussian curves. Approximate locations of rhombomeres r4–r8 are indicated in gray. **(E)** Two minutes of recording in a fish with frequent spontaneous eye movements. In this experiment, NpHR was stimulated four times on the left side after the fish made a saccade. Each stimulation is marked as a vertical red line in the recording and the red color intensity of the line corresponds to the light power used for the stimulation. The relative stimulation intensity (black line) is plotted in addition below the eye traces. Panel **(A)** is modified from Figure 4 of Baier and Scott (2009).

To measure the effects of such instantaneous perturbations, and in turn infer the dynamics around the line attractor, we used fiber optic stimulations (Arrenberg et al., 2009) in behaving transgenic zebrafish (Figures 4A,B, **Figure A7**). Zebrafish are likely to have the same basic oculomotor circuit architecture and physiology as adult goldfish. The zebrafish larvae (5–8 days post-fertilization, dpf) were immobilized in agarose, and the agarose surrounding the eyes was removed. An optic fiber was positioned above the hindbrain to stimulate halorhodopsin (NpHR) or channelrhodopsin (ChR2). The transgenes *Tg(UAS:ChR2(H134R)-mCherry)s1986t* or *Tg(UAS:NpHR-mCherry)s1989t* were driven by the enhancer trap line *Et(E1b:Gal4)s1101t*, resulting in broad expression of ChR2 or NpHR in neurons (Scott et al., 2007; Arrenberg et al., 2009). To localize the zebrafish OI (Miri et al., 2011a) we performed unilateral light stimulations on different rostro-caudal positions of the hindbrain and measured the resulting eye drift magnitudes (Figures 4B–D). We chose short 200 ms stimulations to use the same time scale as burst signals which the position neurons typically integrate and to allow the NpHR-induced hyperpolarization to saturate. Two previous reports (Miri et al., 2011a,b) localized the integrator neurons in rhombomere 7 and 8 of the larval zebrafish, based on the corresponding location in the goldfish, two-photon laser ablations and on optogenetic loss-of-function experiments. In these laser ablation and optogenetic experiments, a single location was tested (rhombomere 7 and 8) and found to have an effect on the integrator performance. Here, we used small diameter optic fiber stimulations (50 μm) to test multiple positions (Figure 4C). The photoactivated volumes were columns of tissue approximately 10–15 cells wide and protruding through the entire dorsoventral extent of the hindbrain, as judged by Kaede photoconversion experiments (**Figure A7**). The maximal effect was observed around 50–150 μm caudal of the Mauthner cells, somewhat more rostral (rhombomere 5, 6, and 7) than in the previous reports (Miri et al., 2011a,b) (Figure 4D, Supplementary Movie 1).

**Figure 5:**
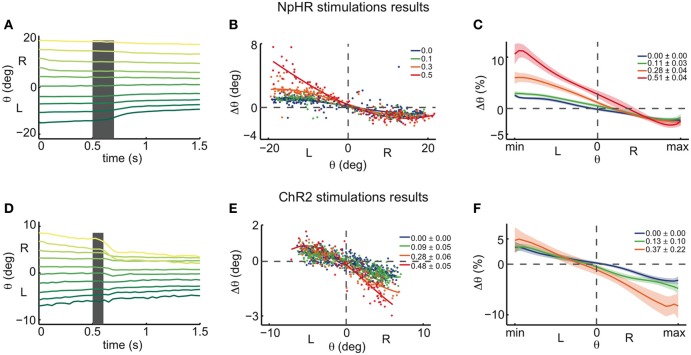
**Halorhodopsin and channelrhodopsin instantaneous stimulations of the left OI: experimental results. (A)** Experimentally recorded eye position traces, averaged across eyes, during NpHR stimulation (0.5 mW). Averages over 96 eye traces (for each eye) of 1.5 s each, distributed across 10 initial eye position bins, in one fish. Same format as in Figure 3C. Stimulation of the left OI elicited movements toward the right in the left range, and small or no movements toward the left in the right range, in agreement with the ILA model predictions from Figure 3C. **(B)** Experimentally recorded eye position changes, Δθ, as a function of initial eye position, θ, averaged across both eyes (see **Figure A6**, for individual eyes). Data are from the same single fish recording as in **(A)** (for each eye, 372 stimulations, 258 control points, 4 intensity bins, over the course of 3 h). Same format as in Figure 3D. The stimulations were binned and colored according to the average stimulation light power in mW for each bin. Spline fits were performed on the binned data to highlight the data trend. **(C)** Spline fit averages across all tested fish (*n* = 24 recordings), same format as in Figure 3D. The motor ranges of all fish were normalized before averaging, and the y-axis indicates the eye position changes Δθ as a proportion of the motor range. Legends indicate the light power averages in mW for each bin and respective standard errors across recordings. The shaded envelopes indicate standard errors. **(D)** Experimentally recorded eye position traces during ChR2 stimulation (between 0.37 and 0.56 mW). Averages over 76 stimulations in one fish. Same format as in Figure 3C. The stimulation elicited movements toward the left in the right range, and small or no movements toward the right in the left range, in contrast with ILA model predictions from Figure 3C. **(E)** Experimentally recorded eye position changes, Δθ, as a function of initial eye position, θ, averaged across both eyes (see **Figure A6** for individual eyes). Data are from the same single fish recording as in **(D)** (for each eye, 523 stimulations, 382 control points, over the course of 3 h). Same format and analysis protocol as in panel **(B)**. **(F)** Spline fit averages across all tested fish (*n* = 19 recordings), same format and analysis protocol as in panel **(C)**.

We first focused on inhibitory (NpHR) perturbations. Unilateral stimulations about 100 μm caudal of the Mauthner cells (rhombomere 7 and 6) resulted in a drift of the eye position following a saccade (Figure 4E). As in the numerical simulations protocol, we computed the changes Δθ in eye position from just before the stimulation to 1 s after the stimulation. For simplicity, all unilateral stimulation results are plotted as left stimulations. Figures 5A,B show the results for a single fish: the eye positions are perturbed strongly in the left range and only weakly in the right range. In the left range, the more eccentric the initial eye position, the higher the elicited change in the eye position toward the right, as indicated by the positive Δθ. Additionally, the magnitude of this change increased with the stimulation intensity. These results become more distinct when averaging over all fish (*n* = 24) (see Figure 5C).

The results of the halorhodopsin activation experiments therefore agree with the ILA model that we used as an example network model in Figures 3A–D. We note two small differences to the predictions of the ILA model. First, even in the absence of NpHR stimulation, the eyes move slowly toward the null position from the whole motor range. Accordingly, the zebrafish eyes are slightly mistuned, whereas the ILA model is not. Second, the eyes show positive perturbation Δθ at the null position, unlike the model predictions (with Δθ = 0 for θ = 0). This difference could be due to uncertainties in the eye position: since the model only predicts positive perturbations, Δθ ≥ 0, any ambiguity in eye position, be it due to uncertainty of the null position (see Materials and Methods), measurement errors, or hysteresis in the system itself, will shift the average perturbation at θ = 0 to a positive value. Altogether, the dynamics of the OI below the line attractor are therefore well-captured by the ILA model (compare Figure 2E). The halorhodopsin inactivation experiments thereby confirm the pharmacological inactivation experiments of Aksay et al. (2007).

### Unilateral optogenetic perturbations: results of ChR2 experiments

Following the same protocol as in the NpHR experiments, we next performed unilateral instantaneous stimulations (100 ms) in animals expressing ChR2 so that one side of the OI was excited (Figures 5D–F). Again, all unilateral stimulations were pooled and plotted as left stimulations. When the initial eye position was in the right range, the perturbations generally caused an eye movement toward the left, i.e., toward the null position, as indicated by the negative Δθ. In this case, we furthermore observed that more eccentric initial eye positions or higher intensity stimulations induced stronger changes in the eye position. On the other hand, when the initial eye position was in the left range, stimulations elicited very small or no changes toward the right after 1 s, when compared to the control case (Figures 5D–F).

**Figure 6:**
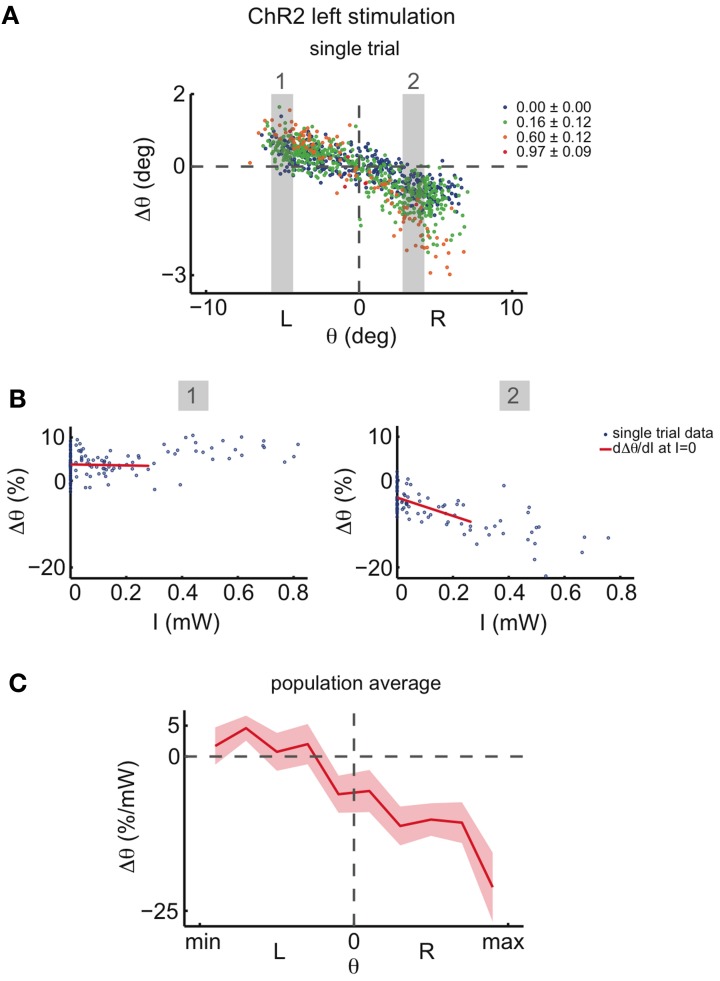
**Effect of ChR2 stimulations at very low light intensities. (A)** Eye position changes Δθ caused by left side ChR2 stimulation vs. eye position previous to stimulation, for one recording, averaged across both eyes. Data were binned and colored according to light power. Legends indicate the stimulation light power average in mW for each bin. The two gray shaded areas highlight the ranges of initial eye positions θ for which the function between Δθ and intensity of stimulation, *I*, is analyzed in **(B)**. **(B)** Δθ vs. intensity of stimulation, *I*, for the two highlighted eye position ranges in **(A)**. In blue are the data points, and in red a linear regression fit for the data in the range 0 mW < *I* < 0.28 mW. The derivative of the function between Δθ and *I* at *I* = 0 is assumed to be the slope of this linear regression fit. **(C)** Averages of the derivatives *d*Δθ/*dI* (*I* = 0) vs. eye position previous to stimulation across all tested fish (*n* = 19 recordings). The plot envelopes indicate standard errors.

For very high ChR2 stimulation intensities, we additionally noted eye movements toward the null position when the initial eye position was on the same side as the stimulation (ipsiversive eye positions; **Figure A6**). Since we are interested in inferring the dynamics in the immediate vicinity of the line attractor, we linearly regressed the magnitude of the drift at very low stimulation intensities (Figure 6A). In agreement with the data for medium intensities (Figure 5F), low intensity stimulation induces eye movements mostly during contraversive eye positions (when the initial eye position is on the other side as the stimulation; Figures 6B,C).

The results of the channelrhodopsin activation experiments therefore invalidate the ILA model, as we find that weak excitatory unilateral perturbations of the left OI induce centripetal (toward the null position) and not centrifugal eye movements. Furthermore, we find the strongest effect on the side contralateral to the perturbation, and almost no effect on the ipsilateral side. We note that the perturbations resulting from experimental excitation and inhibition are approximately point-symmetric with respect to the central eye position, (θ = 0, Δθ = 0), and not reflection-symmetric with respect to Δθ = 0, as proposed by the ILA model.

### The OI dynamics around the line attractor

**Figure 7:**
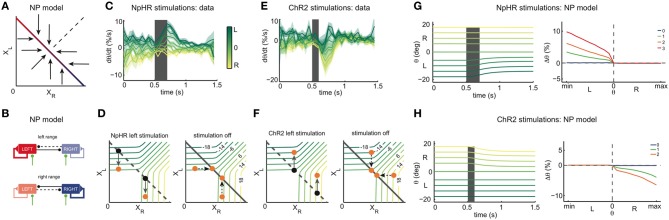
**Inference of the OI dynamics. (A)** State space of the model inferred from the NpHR and ChR2 instantaneous stimulations: the NP model. In the NP model, the dynamics below the line attractor are orthogonal to the dynamics above the line attractor. **(B)** Effective connectivity of the NP model. In the left range (top), the left network receives stronger self-excitatory and weaker cross-inhibitory inputs than the right network. For low activity of the right network, the left network sustains its firing, whereas for high activity of the right network, the activity of the left network is decreased. In the right range (bottom), the inputs and dynamics are reversed. Overall, cross-inhibition is very weak. **(C)** Average across all experimental recordings of the derivatives of the eye position traces during left side NpHR stimulation (light power between 0 and 0.8 mW). Eye position derivatives are measured as a proportion of the motor range. Colors indicate the initial eye position (before the stimulation). The shaded envelopes indicate standard errors. For initial eye positions in the right half of the motor range (yellow traces), the left side stimulation leads to either small positive deflections of the eye position (i.e., toward the right), or null deflections. In contrast, for initial eye positions in the left half of the motor range (green traces), the left stimulation leads to positive derivatives, i.e., strong rightward eye movements. **(D)** State space interpretation of the transient dynamics in **(C)**. Left: dynamics during left inhibitory stimulation. The left population activity decreases, while the right population activity is largely unaffected. Right: relaxation to line attractor after stimulation. In the right range, the system relaxes to positions close to the position previous to stimulation. **(E)** Average across all ChR2 trials (light power between 0 and 0.28 mW) of the derivatives of the eye traces. For initial eye positions in the right half of the motor range (yellow traces), the left stimulation leads to negative deflections of the eye position, i.e., toward the left. In contrast, for initial eye positions in the left half of the motor range (green traces), the left stimulation leads to biphasic eye movements, first negative derivatives (i.e., leftwards) then positive derivatives (i.e., rightwards). **(F)** State space interpretation of the transient dynamics in **(E)**. Left: dynamics during left excitatory stimulation. The left population activity increases, while the right population activity is largely unaffected. Right: relaxation to line attractor after stimulation. **(G)** NpHR stimulation of the NP model. Left: simulated eye position traces during left stimulation (gray shaded period), with same format as Figure 5A. Only initial eye positions in the left half of the motor range are affected by the stimulation. Right: NP model predictions for the eye position changes, Δθ, as a function of the initial eye position, θ. **(H)** ChR2 stimulation of the NP model. Left: simulated eye position traces during left stimulation (gray shaded period), with same format as Figure 5D. Stimulation causes eye position changes only in the right half of the motor range. Right: NP model predictions for the eye position changes, Δθ, as a function of the initial eye position, θ. In the right panels of **(G,H)**, averages were computed over 4000 stimulations in each of 21 eye positions, for each intensity of stimulation.

The results from the NpHR and ChR2 experiments provide us with the means to infer the OI dynamics around the line attractor. Since the inhibitory stimulations yield results similar to those predicted by the ILA model (compare Figure 3D and Figure 5C), we conclude that the dynamics below the line attractor are similar to those of the ILA model (Figure 2E). To obtain the observed point-symmetry of the perturbations (Figures 5C,F), however, we need to assume that the dynamics above the line attractor are roughly orthogonal to the dynamics below. Consequently, our experimental results suggest a model with dynamics as shown in Figure 7A. Here, the dynamics outside the line attractor are organized around a central point, corresponding to the null position of the eyes (the “null position” or NP model). Although Figure 7A illustrates the overall flow of the trajectories toward the line, we note that our line attractors are composed of individual fixed points, such that the fine-scale dynamics change in the vicinity of the line (see **Figure A4C**). Although the fine-scale dynamics predict small changes toward the left in the left range after ChR2 stimulation, these small changes do not appear in the predictions if we assume that the system is noisy.

These conclusions are further supported when we analyze the transient dynamics of the experimental eye movements around the NpHR or ChR2 stimulation period (Figures 7C–F). If the initial eye positions are in the left range, the NpHR stimulation generates monotonic eye movements toward the right, as indicated by the transient, positive derivatives of the eye movements (green traces in Figure 7C). If the initial eye positions are in the right range, the NpHR stimulation leaves them essentially unaffected. This contrasts with the ChR2 stimulations which transiently affect all eye positions. For initial eye positions in the left range, the stimulation causes transiently biphasic eye movements, as indicated by the negative, then positive derivatives of the eye movements (green traces in Figure 7E). In other words, the eye is briefly moved to a more eccentric position, before relaxing back to its original position. For initial eye positions in the right range, we observe an overall negative derivative of the eye movement, generating a centripetal eye position drift (yellow traces in Figure 7E).

These transient dynamics match with the population activity dynamics predicted by the NP model (Figures 7D,F). Let us focus on the excitatory perturbations. When the left OI is excited, the left population activity *X*_*L*_ increases in the whole motor range (Figure 7F). In the relaxation phase, if the initial eye position is in the left range, the system counterbalances the left stimulation by decreasing *X*_*L*_ without changing *X*_*R*_. Accordingly, eye movements are biphasic and altogether eye position changes only marginally. In the right motor range, the right population activity decreases in the relaxation phase, while the left population activity stays intact, thus causing centripetal (to the null position) eye movements (Figure 7F).

Besides explaining the perturbation data well (Figures 7G,H), the resulting NP model is also in better agreement with the natural leakiness of the OI, i.e., the slow drift in eye positions that we observe even in the absence of perturbations (Figures 5C,F). First, we note that many (random) mistunings of the synaptic parameters will automatically lead to leaky eye positions. Second, even in the perfectly tuned NP model, the slow centripetal drift will emerge due to the presence of noise in the neuron's firing. Since any internally generated noise in the OI will have similar (if smaller) effects than our perturbations, such noise will cause slow centripetal, and thereby leaky eye movements (see **Figure A4D**).

### Stimulation of afferents to the integrator

While the dynamics that we inferred through the experiments seem perfectly reasonable, the applicability of the NP model to the OI hinges on the correctness of our experiments. One particular concern is that the limits of the integrator cells in the larval zebrafish are not well-established. Hence, we cannot rule out that we may be stimulating efferent motor neurons or afferent inputs (for instance, vestibular neurons) to the integrator. Since stimulation of motor neurons should not lead to persistent eye position changes, we ignore the potential stimulation of those neurons. However, stimulating afferent inputs to the integrator, in addition to the integrator cells remains a possibility. Using the modeling framework, we show below that accidental stimulation of these afferent inputs does not invalidate our conclusions.

**Figure 8:**
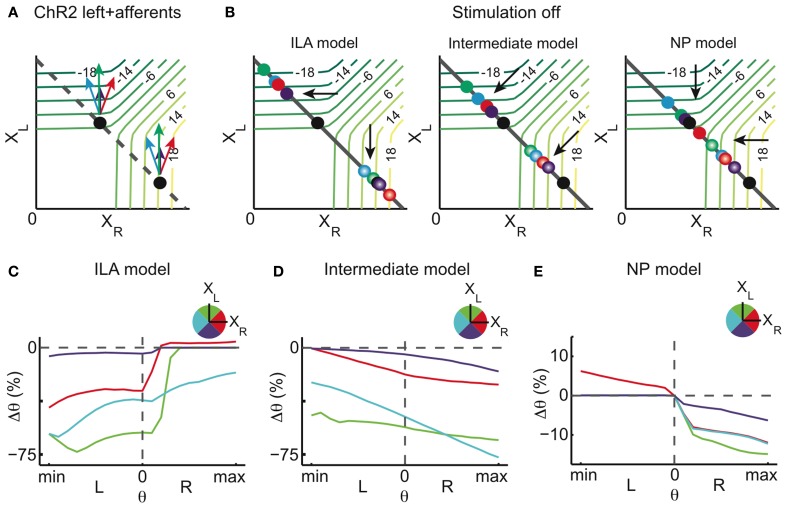
**Stimulation of afferents to the integrator (model)**. Potential ChR2 stimulation of afferents to the left and right half of the integrator in addition to direct ChR2 stimulation of the left OI. **(A)** Stimulation of afferents to the left OI changes the overall magnitude of the stimulation. When affecting excitatory afferent neurons, the stimulation magnitude is increased (green arrows), when affecting inhibitory afferent neurons, it is decreased (violet arrows). When afferents to the right OI are stimulated, an orthogonal component is added to the overall stimulation effect. Additional stimulation of inhibitory afferent neurons pulls the perturbation to smaller values of *X*_*R*_ (blue arrow) whereas additional stimulation of excitatory afferent neurons increases the right population activity (red arrow). **(B)** Relaxation after stimulation for three different models: ILA model, intermediate model, and NP model. The final state of the system is represented by dots, colored according to the respective afferent stimulation. **(C–E)** Eye position changes Δθ caused by direct and afferent stimulation (afferent stimulation has half the magnitude of the direct integrator stimulation) vs. eye position previous to stimulation for the three different models, where color indicates the direction of afferent stimulation in the state space. Neither the ILA model nor the intermediate model lead to results similar to the data: the ILA model mainly leads to centrifugal movements on the ipsiversive side regardless of the type of afferent stimulation; the intermediate model leads to changes toward the left in most of the motor range, for all afferent stimulations. Only the NP model leads to results similar to the data, and especially so when the net afferent stimulation leads to contralateral excitation (red curve). For each intensity of stimulation, the respective plot corresponds to averages over 4000 stimulations in each of 21 eye positions.

We distinguish four possible scenarios for afferent stimulation (Figure 8A): in the first two scenarios, afferent stimulation leads to either ipsilateral excitation or inhibition of the integrator cells (violet and green arrows), and in that case our overall stimulation direction does not change in the synaptic population output space. In the other two scenarios, the afferent stimulation leads to either contralateral excitation or inhibition of integrator cells (red and blue arrows), therefore introducing a component to the stimulation which is orthogonal to the pure integrator stimulation.

For a cross-inhibitory architecture, the dynamics above the line attractor are either as in the ILA model (Figure 2E), as in the NP model (Figure 7A), or somewhere in between (in that case, the dynamics are roughly perpendicular to the line attractor, as in Figure 2D). We simulated all four afferent stimulation scenarios for each of the three models, and assumed that afferent stimulation has half the magnitude of the direct integrator stimulation. As can be seen in Figures 8B–E, neither the ILA model nor the intermediate model produce results similar to the data (regardless of the type of afferent stimulation). Only the NP model leads to the results observed in the data.

Furthermore, the NP model provides an explanation for the effect of the high intensity stimulation mentioned earlier (compare **Figure A6**): if at high intensity, unilateral ChR2 stimulation also excited the contralateral side via light scattering, then the NP model would indeed predict centripetal eye drifts on the ipsilateral side (Figure 8E, red curve).

### Connectivity in the integrator

In conclusion, the NP model is the only candidate model within our theoretical framework that agrees with all aspects of the data. Most importantly, these dynamics suggest a different connectivity than implied by the previously proposed models. The respective, effective connectivities for the NP model are shown in Figure 7B. The dynamics are mediated by weak mutual inhibition and a self-excitation whose effective strength depends on eye position, suggesting that both sides are almost, albeit not completely, decoupled, in line with previous findings in the goldfish (Debowy and Baker, 2011). To keep the self-excitation in check, external (e.g. vestibular) inputs to this model are inhibitory. A detailed description of the implementation of the network models can be found in the Materials and Methods.

## Discussion

### Summary

We showed that a whole range of network models could account for the set of electrophysiological features that have been measured in OIs across animals of different species. These models differ from each other by the relative strength of self-excitation and mutual inhibition, and the respective dynamics prevailing in the population activity state space outside of the line attractor. To test these models and experimentally unveil the dynamics of the OI, we performed optogenetic perturbations in the larval zebrafish. Using the silencer NpHR, we found that unilateral light stimulation induced eye movements back to the midpoint, if the eye position prior to the stimulation was on the same side as the stimulation. The experimental results for NpHR in larval zebrafish were in accordance with results from goldfish using inactivation with lidocaine (Aksay et al., 2007), therefore corroborating the previously proposed ILA population dynamics. However, we found that unilateral ChR2 stimulations did not have the opposite effect to NpHR stimulations predicted by the ILA dynamics, i.e., centrifugal (away from the null position) eye movements on the stimulation side (ipsiversive eye positions). Instead, ChR2 stimulations had a centripetal (toward the null positions) effect on eye positions on the side opposite to the stimulation (contraversive eye positions).

Hence, perturbations always tend to drive the eye positions toward the midpoint, indicating that this point is the focus of the OI dynamics. Consequently, we inferred the dynamics around the line attractor from these experiments, and named the resulting model the null-position or NP model. This model suggests an OI architecture with strong self-excitation and weak cross-inhibition. Only a specific combination of excitation on one side and inhibition on the other will lead to actual changes in the position signal as required in saccadic eye movements (e.g., movements from one range to the opposite range) (Gonçalves, 2012). Interestingly, our results can at least in part explain the findings in pharmacological experiments where glutamate or GABA agonists were injected in the OI. Both an increase in excitation as well as an increase in inhibition resulted in centripetal eye movements, much as in our experiments (Arnold et al., 1999).

### Experimental features and limitations

In the presented study we show how optogenetic experiments can be combined with modeling to infer the dynamics of a neural circuit module for integration. In the last few years, the field of optogenetics has provided a powerful set of techniques to perform gain- and loss-of-function experiments (reviewed in Zhang et al., 2007a; Luo et al., 2008; Fenno et al., 2011) and has been applied to zebrafish (Szobota et al., 2007; Douglass et al., 2008; Arrenberg et al., 2009; Baier and Scott, 2009; Zhu et al., 2009; Schoonheim et al., 2010). A fundamental problem in interpreting the effects of optogenetic stimulations is that a system's response is a combination of the stimulation magnitude and the intrinsic network dynamics. Indeed, the eye movements induced through the optogenetic perturbations depended on both the light-intensity, i.e., the strength of stimulation, and on the eye position, i.e., the internal state of the system prior to stimulation. To understand these interdependencies, we relied on network modeling (Seung, 1996; Seung et al., 2000; Goldman et al., 2003; Eliasmith, 2005; Aksay et al., 2007). In turn, the mismatches between the model predictions and the experimental results allowed us to constrain the class of feasible network models and thereby improve our understanding of the OI. This general approach illustrates the importance of the internal state of a system during a perturbation. Wherever this internal state is at least partially known, optogenetic perturbations can provide useful clues toward the underlying network dynamics.

In our experiments we made use of a Gal4 driver line that drove strong expression broadly in neurons. While local stimulation in this line led to marked behavioral changes, the manipulation was not specific to the neural integrator. The neural integrator in larval zebrafish is distributed across approximately 150 μm in the hindbrain of the larval zebrafish (Miri et al., 2011a), with non-integrator neurons interspersed between the cells with position signals. However, our results are not easily explained by a stimulation of these non-integrator neurons. First, both NpHR and ChR2 stimulation induce stable and persistent changes in eye position. This persistent change makes an influence of the motoneurons that lie in close rostral proximity to the integrator unlikely. Exclusive motoneuron stimulation should cause the eyes to move back to the original position immediately after stimulation offset, an effect we did not observe. Second, we may have stimulated cells which project to the integrator such as the saccade-generating neurons. However, saccade generating neurons are only active during saccades and unilateral stimulation is therefore expected to only change the saccade frequency (Schoonheim et al., 2010) and have no effect in-between saccades. Nevertheless, a low level stimulation of the saccade-generating neurons could have occurred without the generation of a measurable saccade: in that case, given that saccade-generating neurons excite the ipsilateral OI and inhibit the contralateral OI, a perturbation of these neurons would cause an indirect stimulation of the integrator neurons roughly in the same direction as the direct integrator stimulation, therefore not invalidating the interpretation of our results. Third, we may have affected some of the vestibular inputs to the OI. However, even in this scenario, our conclusions about the integrator dynamics hold up. Since vestibular inputs are included in the network models, we can simply simulate their accidental stimulation. As shown in Figure 8, accidental stimulation offsets the magnitude of induced eye drifts, but overall does not alter their eye position dependence. Within the range of models considered, the data can therefore only be explained by the NP model, but not by the other models.

We have stimulated excitatory and inhibitory integrator cells at the same time, which could potentially lead to unexpected network effects, e.g., due to induced imbalances of excitation and inhibition within the network. However, several observations support our interpretation of the data. First, electrophysiological recordings in the hindbrain of the same zebrafish lines used in this study suggest that more than 80% of NpHR expressing cells were significantly silenced during illumination and more than 90% of ChR2 expressing cells showed an increase in firing rate upon illumination (Arrenberg et al., 2009). Second, NpHR stimulation results are in agreement with the pharmacological inactivations in the goldfish integrator (Aksay et al., 2007), therefore confirming the inhibitory nature of the NpHR stimulations on the integrator. Third, ChR2 stimulation leads to different results than NpHR stimulation, which is proper inhibition. Fourth, both ChR2 and NpHR experiments were performed with the same Gal4 driver line. Therefore, if ChR2 stimulation led to net inhibition of the integrator, then NpHR stimulation would lead to excitation of the integrator which is ruled out based on the second observation. While these results suggest that optogenetic manipulations of the neural integrator changed the network activity in the expected direction, future zebrafish lines, e.g., with specificity for excitatory or inhibitory neurons, will facilitate the dissection of this circuit.

One could hypothesize that the effect of ChR2 stimulation saturates or reverses (depolarization block, Kleinlogel et al., 2011) with increasing stimulation intensities or for highly active cells. Our previous electrophysiological recordings provided no evidence for such an effect (Arrenberg et al., 2009, **Figure A9**). More importantly, this possibility is not supported by the eye movement data in the range of intensities analyzed, since the eye movements scale linearly with the stimulation light intensity (Figure 6B). Also, the effect does not reverse at high light intensities (**Figure A6**). As a final note, the modeling framework already assumes that the synapses of highly active neurons are saturated, so that, at least within the model, ChR2 stimulation does not affect these neurons.

In this study, we deliberately focused on the dynamics in the neighborhood of the stable eye position states. Consequently, our analysis was restricted to stimulation with low or medium light intensities. The NP model, however, makes predictions for any stimulation intensity, opening the question of what happens when the stimulation intensity is increased. As shown in the **Figure A6**, for high NpHR stimulation intensities, we additionally found small centripetal movements when the eye position was on the side opposite to the stimulation. For high ChR2 stimulations, we found centripetal eye movements when the eyes were on the same side as the stimulation. In the NP model, this would require a change in the dynamics far away from the line attractor, requiring the arrows to bend further toward the midline. While it seems unlikely that the effects at higher light intensities can be explained through scattering of light into the other hemisphere (data not shown), the effect could potentially be explained through strong stimulation of vestibular inputs. Furthermore, we notice that strong ChR2 stimulation could synchronize the activities of cells, which may have a range of effects, including complete shutting down of persistent activity (Dipoppa, 2012). We therefore refrained from including these observations in the model.

### Model features, limitations, and predictions

Integrators are ubiquitous in the brain and are involved in several important computations. For instance, in decision-making tasks requiring sensory integrations, neurons in the lateral intraparietal cortex behave similar to integrators (Gold and Shadlen, 2001; Wong et al., 2007). In working memory tasks, neurons in the prefrontal cortex exhibit almost linear dynamics during the times in which an animal needs to remember a stimulus, similar to integrators operating in several dimensions (Singh and Eliasmith, 2006; Machens et al., 2010). In the head direction system, a head velocity signal is integrated into head position (Zhang, 1996).

In previous line attractor models, it has generally been assumed that noise causes random drift along the line (Seung, 1996). While this is true in models with orthogonal dynamics around the line (such as the model illustrated in Figure 2D), in the case of the NP model the relaxation to the line has a preferred direction, therefore causing a systematic drift toward the null position. The term “line attractor” for the NP model is therefore strictly only valid in the limit of vanishing noise. For large noise levels, the model shows flow toward the central eye position with equivalent speed from every point in the state space. Hence, one could interpret the NP model as suggesting that the OI operates like a single fixed point, and not a line attractor, as extensively suggested in previous literature. However, for large noise levels, we can re-tune the NP model to recover the NP dynamics in the proximity to the line by implementing stronger cross-inhibition (simulations not shown). In any case, random perturbations of the NP model (such as noise) are unlikely to cause a centrifugal drift of the eye position.

Given the centripetal drift suggested by the NP model, we hypothesize that the OI features dynamics with a higher degree of built-in “safety” than previously thought. The OI has been observed to be leaky on longer time scales, both in goldfish and zebrafish (Mensh et al., 2004; Miri et al., 2011a). This leakiness may be a behaviorally advantageous feature, since, by bringing the system to the central position by default, it enables the relaxation of the eye muscles. Yet even higher brain systems may rely on such a built-in leakiness. In working memory tasks that employ graded persistent activity (Machens et al., 2005), for instance, a tendency to drift toward the central point while memorizing a sensory stimulus could explain the psychophysical errors that are known as contraction bias (Ashourian and Loewenstein, 2011).

While this built-in “safety” may help against noise in the system, it does not solve the fine-tuning problem, i.e., the instability of the line attractor against perturbations in the synaptic weights in the network. Indeed, this fine-tuning problem is a separate problem, somewhat orthogonal to the problems that we have investigated here, for which several solutions have been proposed (Koulakov et al., 2002; Goldman et al., 2003; Moreau and Sontag, 2003).

While we here have assumed that neural integration in the oculomotor system is generated through precise recurrent feedback in a neural circuit, in previous literature single-cell mechanisms have been put forward to explain neural integration observed in multiple areas in the brain. In particular, following an experimental demonstration of integration in individual cells from the entorhinal cortex (Egorov et al., 2002), a body of theoretical work has proposed several biophysical mechanisms which could underlie single cell integration, dispensing synaptic feedback (Loewenstein and Sompolinsky, 2003; Fransén et al., 2006). In the OI, unilateral disruption of the connectivity leads to neural activity drifts with time constants which are typically above 1 s (Pastor et al., 1994; Aksay et al., 2007), suggesting that single-cell mechanisms possibly play a role in the process of integration. However, single-cell mechanisms remain largely uncharacterized in the integrator, and therefore we here have followed the network mechanisms hypothesis as in previous studies of this system (Seung, 1996; Aksay et al., 2007). The contribution of single-cell mechanisms to the slow dynamics in the integrator is a challenge for future research.

We also note that our network model is a rate-model, in which the activities of individual cells are described by rates rather than precise spike times. Although we lose biophysical realism with this type of model, we gain analytical tractability, a very useful asset in interpreting experimental results within a theoretical framework, and in constructing models in accordance with data. Since the position cells exhibit persistent activity with regular firing (Aksay et al., 2003), temporal averages of spiking events are a good qualitative description of the system. Nevertheless, an equivalent model with spiking neurons could be built as shown in Seung et al. (2000), Eliasmith (2005).

Given the weak mutual-inhibition, the NP model behaves close to a system with a plane of stable fixed points (see Figure 2F), and shows slow dynamics around the line. We note that the relaxation of the eye positions after stimulation is indeed slow (on the order of 200 ms). However, these slow dynamics could be reflecting the dynamics of the muscle physics rather than the slow dynamics of the integrator. Future work should show whether such slow dynamics can indeed be observed.

Our network model features multiple stable fixed points, which suggests that the eye positions corresponding to these fixed points should be held comparatively longer than eye positions in-between the fixed points. However, in our data, the system seems to visit a range of eye positions in a homogeneous way (both during NpHR stimulation and during spontaneous, slow eye position decay), which contrasts with the prediction of our model. Such homogeneity could be due to external factors to the integrator, such as small saccadic commands causing smooth eye movement fluctuations, or the dynamics of the motor neurons and muscles. Nevertheless, the homogeneity found in the data challenges our hypothesis of discrete fixed points in the integrator and suggests further studies to elucidate this question.

Our modeling framework assumes a specific mapping from network activity to eye position, based on the difference between the tuning curves of position neurons and motor neurons (see Materials and Methods). Although this assumption is essential in our framework to account for the unilateral inactivation results in Aksay et al. (2007), one could relax it and assume linearity between population synaptic outputs and eye position by introducing high synaptic thresholds in the same fashion as in Aksay et al. (2007). However, we believe that the non-linear mapping is most likely present in the system and should be included in future modeling studies of the oculomotor system.

In a recent study (Miri et al., 2011a), zebrafish position neurons were shown to have variable timescales of integration, so that the associated relaxation time constants varied across neurons over one order of magnitude. This suggests that the dynamics of the OI could be high-dimensional (on the order of the number of neurons), in contrast with our line attractor model, which is implemented with homogeneous time constants across neurons and has low (2D) dimensional dynamics. Given that in this study we are interested in the dynamics of the population activities, the details of single-cell time constants do not affect our conclusions. In the future, it will be interesting to perform optogenetic stimulations and at the same time measure the activities of the position cells to explore the full state space, and realize the dimensionality of the system's dynamics.

Our models belong to a series of works suggesting that the OI builds up a line attractor by a balance between neural saturation and progressive recruitment of neurons to compensate such saturation (Seung, 1996; Seung et al., 2000; Aksay et al., 2007). Future work should specifically target the validity of this assumption as it is crucial for the whole modeling framework. Specifically, this assumption predicts that neurons have no responsibility or influence on eye positions that are below their firing threshold or that are sufficiently above that threshold (when they run into saturation). This prediction could e.g., be tested with single-cell ChR2 stimulation. Excitatory stimulation of different cells would then lead to movements in different eye ranges, enhancing the fact that different neurons are responsible for different stable activities on the motor range (Gonçalves, 2012). Consequently, combining single-cell optogenetics with the framework here designed has the potential to provide even deeper insights into the detailed structure of the OI in the future.

## Materials and methods

### Experiments

#### Animals

For all experiments, we used zebrafish larvae between the age of 5 and 8 dpf. Animals were transgenic for a combination of the following transgenes: *Et(E1b:Gal4)s1101t, Tg(UAS:NpHR-mCherry)s1989t, Tg(UAS:Kaede)s1999t, Tg(UAS:ChR2-mCherry)s1986t*. In addition, the larvae were mutant for the *mitfa*/*nacre* gene (*mitfa*^*s*170^, *mitfa*^*s*184^, or *mitfa*^*b*692^ alleles), which rendered the skin transparent and facilitated fiber optic stimulation as well as eye position detection. Siblings that did not express NpHR or ChR2 served as control groups and are labeled wt here. Adult fish were either transgenic for *Et(E1b:Gal4)s1101t* or for the optogenetic responders, since keeping optogenetic expressors in the s1101t line would have resulted in variegation of the expression. Embryos/larvae were raised in the dark and not fed. For each experiment, about 4 clutches were screened and the strongest expressors were kept.

Many Gal4s1101t/UAS:NpHR expressing larvae had non-inflated swim bladders or showed only infrequent eye movements. For this reason, each mounted larva was observed for 1 min under a stereoscope and only larvae that showed saccades in both directions and good peripheral eye fixations were used for the experiments. This way, only the best behaving 20% of the mounted larvae were used. Control larvae were screened the same way, although a higher percentage of larvae could be used for experiments. The screened NpHR expressing and non-expressing larvae had similar eye drift rates in the absence of stimulation (see Figure 4 in Miri et al., 2011a). The magnitudes of the induced eye position drifts were somewhat variable between animals stimulated at the same position, which we attribute to the expression level/variegation variability between animals. For example, in one animal in **Figure A8**, we noted a patch of cells in which NpHR expression was absent, which resulted in a much reduced effect on eye position (see points [0.08, 86 μm] and [0.15, 86 μm] for the left and the right eye in the ipsi stim. condition). We excluded 5 animals from the analysis in Figure 5F, since the induced eye position changes were much smaller than in the majority of animals.

#### Mounting

Larvae were mounted in a drop of low-melting agarose (1.6%) in a petri dish (35 mm diameter). A platinum wire (100 μm in diameter) glued to a pasteur pipette was used to flatten the liquid agarose drop by moving the wire at the perimeter of the drop and thus increasing the agarose-covered area in the dish, so that the height of the liquid approximately matched the height of the larva. This step took 10 s and ensured that the optic fiber could be placed close to the skin in the experiment. Next, we used the wire to position the larva dorsal side up. The agarose solidified for 5–10 min and egg water was added. A second platinum wire (100 μm) that was flattened at the tip and bend 70° about 2 mm from the tip was used to make agarose incisions, moving the platinum wire sideways so that the flattened part acted like a knife. Sometimes the agarose would lose its adhesion and we found that using a fresh petri dish for every fish ameliorated this problem. Also, the fish sometimes managed to escape and we found that inserting the platinum wire directly at the eye and moving it outwards worked better than the other way round. Two blocks surrounding the eyes were cut and the flat side of the platinum wire was now used to scoop the blocks out. The stereoscope backlight was set at an angle (dark field) to visualize the cut agarose and make sure that the agarose surrounding the eyes was completely removed. After 3 min of rest, the frequency of eye movements was observed (see Animals). To minimize water evaporation, a dish lid was placed on the petri dish in some of the recordings. The dish lid had a 1 cm diameter hole to allow for the placement of the optic fiber.

#### Laser stimulation

For laser stimulations, we used an AOTF to couple lasers of three different wavelengths (633, 561, 488 nm) into a multimode optic fiber. We made use of the laser system of a disassembled confocal microscope. For UV photoconversions, we manually switched the stimulation fiber to a fiber coupled UV laser (355 nm). Fiber preparations were described previously (Arrenberg et al., 2009). The AOTF was used to modulate the laser intensity by providing an analog voltage from the DAQ device connected to the computer. For some experiments we used a multi-laser system from Ikecool (Anaheim, CA), however the analog modulation was not good enough to precisely control light intensity on a millisecond-basis (significant baseline light at 0 Volts and the intensity was not stable enough after a switch from 0 Volts to almost full power). We recommend the inclined scientist to rather buy a system from Toptica (Munich, Germany) or Omicron (Rodgau-Dudenhofen, Germany). The optic fiber (including Thorlabs' FT030 protective tubing) was placed in a glass pipette with an angled tip. The pipette was mounted on a fine micromanipulator and the fiber was positioned over the hindbrain. While x-y position could be judged easily by looking through the stereoscope, the z-positioning was more difficult. In cases in which the z-position was unclear, we used the fine micro-manipulation to lower the fiber tip slowly until (a) the mechanic strain on the agarose moved the skin of the fish slightly or (b) until the fish startled. We then moved the fiber back up by a small distance (≈20 μm).

#### Experimental setup

The larva was placed under a stereoscope and a custom-built LED array was used as a backlight. White LED light was used to position the animals and IR LED light (850 nm) to image the head with a CCD camera (up to 60 Hz, TheImagingSource, Bremen, Germany). During the experiment, dim room lights were kept on and sometimes a weak white backlight was used in addition so that the experiments were performed under low light intensity and low contrast conditions. We did not record in the dark, because wildtype animals showed a behavioral response to the stimulation light in the dark, which was much reduced or absent in the presence of the backlight (Miri et al., 2011a) (the larvae probably see some of the scattered stimulation light). We note that larval post-saccadic eye fixations were somewhat leakier in the dark (when compared to low light conditions), suggesting that larvae were using visual feedback to improve ocular stability. However, this effect was small in comparison to eye position changes induced by medium light intensities, so that we are confident to be measuring the integrator performance in our experiments. A custom-written LabVIEW program (National Instruments, Austin, TX) was used to image, record angular eye positions, and to trigger stimulations via a NI USB DAQ box. Images were acquired at 30–45 Hz and particle analysis (NI Vision) was used to detect eye positions and write them to a text file (together with the stimulation time points) for later analysis. Eye positions were measured and plotted in real time and fiber optic stimulations were triggered based on them. In most experiments a single 200 ms stimulation of constant intensity was delivered automatically 1 s after the eyes had made a saccade. In some experiments four consecutive stimulations were delivered for each saccade (1, 3, 5, and 7 s after the saccade). The stimulation intensities were chosen randomly: we either used five fixed intensities or the computer picked values from an exponential distribution of intensities that biased toward smaller intensities. No stimulation was applied after every third saccade, and these periods served as our control trials. Recordings typically lasted for at least 30 min and up to 12 h. The LabVIEW program can be requested from the authors.

### Data analysis

The files containing eye position trace and stimulation data were analyzed with custom algorithms using MATLAB. We only analyzed the first 3 h of each experiment, since in some recordings (without dish lid) water evaporation affected the camera focus and thereby the correct detection of eye position angles. The induced eye movements were measured as the change in eye position from stimulation start to 1 s after stimulation start. Most induced eye movements consisted of a single monotonic drift that was completed within 400 ms after stimulation. However, a 1 s interval was chosen to account for potential variations of the duration of the post-stimulation drift. The eye position change, averaged across left and right eye, was then plotted against the initial eye position, averaged across eyes. The results from each fish were fitted by a cubic smoothing spline with boundary second derivatives equal to zero, using the spline fit-package developed for MATLAB by Jonas Lundgren. This fitting procedure was repeated for several stimulation intensity bins. A population plot was generated by averaging the spline fits of all fish, for each intensity bin. We observed some variability of motor ranges across fish, especially in animals expressing ChR2, which tended to have a smaller motor range (average motor range and respective standard deviations in NpHR animals: [−20.0° ± 5°; 18.7° ± 4.4°]; in ChR2 animals [−13.7° ± 3.9°; 13° ± 3.5°]). Therefore, before averaging across animals, we normalized all motor ranges to generate a smooth population plot. In some fish, there was an undersampling of events at the eccentric eye positions, and therefore we removed the 5% most eccentric events on the right side and on the left side before averaging across animals. In several fish, some intensity bins did not span the full motor range, and we extrapolated the corresponding splines. To account for the uncertainty of the extrapolation, we performed a weighted average over fish for eccentric eye positions, with weights that decreased as the extrapolation distance increased.

The null-position of the eyes was defined as the average of the extreme eye positions. Note that this definition only works well for eyes which explore eye positions in both directions equally well, which was not the case for every fish. However, in our hands this was the best definition since other definitions (median, average, fixed at +8°/−8°) were more subjective or decreased the across-fish consistency of the results. An animal's distribution of post-saccadic eye positions was often bimodal, since the animal explored the peripheral positions more frequently. In some experiments, we stimulated multiple times (1, 3, 5, and 7 s) after a single post-saccadic fixation in order to access the full scope of eye positions.

The location of stimulation was in caudal proximity to the previously identified region containing saccade generating neurons (Schoonheim et al., 2010), and in a fraction of the events, the stimulation induced a saccade (more frequently for ChR2 stimulations than for NpHR). Since this study focuses on the performance of the integrator, we did not include these events in our analysis. In Figure 5, these were excluded by a velocity threshold (>20°/s). In Figure 4D, the data points result from eye velocity vs. eye position fits (linear regression through the origin, see **Figure A8**), and correspond to the inverse of the eye position drift time constant at a specific hindbrain location. For each fit, only the middle 70% of the eye velocity/eye position data points were fitted, thereby excluding outliers caused by saccades. For each eye, the eye drift was normalized across hindbrain positions. Each data point in Figure 4D corresponds to one fish (average of the normalized left and right eye, for individual eyes see **Figure A8**) and hindbrain position. The data points for Figure 4D (eye drift vs. hindbrain position) were then fitted by gaussian functions. For the Gaussian fit functions in Figure 4D, the mean was restricted to the interval [0; 200]. This modification was made because for approximately flat distributions (control data points ipsi ctrl, contra stim, contra ctrl) peripheral data points sometimes caused Gaussian fits with means far away from the tested hindbrain region.

### Theoretical framework: network model

In previous work (Gonçalves, 2012), we show how to build a line attractor model accounting for the main findings in the oculomotor integrator, using basic design principles that have previously been suggested in the literature (Seung, 1996; Eliasmith, 2005; Machens and Brody, 2008). We use these principles to clarify how the network connectivity is related to the tuning curve properties. Here, we summarize the main features of the model developed in Gonçalves (2012).

We start by describing the assumptions and simplifications underlying the network model. Most of these assumptions are adapted from the previous literature and are based either on arguments of plausibility and simplicity or on observations about the oculomotor integrator (Seung, 1996; Aksay et al., 2000; Seung et al., 2000; Aksay et al., 2007; Machens and Brody, 2008).

*Assumption 1 (Two opposing populations)*. We assume that the dynamics are controlled by two population variables, *X*_*R*_ and *X*_*L*_, one for each side. These variables shall represent the effect one population has on the postsynaptic currents of neurons in the other population; a specific definition will follow below. We assume that in its normal working regime, these two “synaptic” population activities oppose each other, so that, as the activity in one population grows, that of the other drops. For simplicity we therefore assume that

(1) $$\beta =X_{R}+X_{L}$$

where β is a constant value.

*Assumption 2 (Eye position)*. We furthermore assume that within this working regime (but not outside of it) the eye position can be read out as

(2) $$\theta =X_{R}-X_{L}$$

where right range positions are defined as positive eye positions, and left range positions as negative.

*Assumption 3 (Firing rates)*. We furthermore assume that each neuron's firing rate in the stationary state is determined by its excitatory input from the same population, scaled by a weight *a*_*i*_, the inhibitory input from the opposite population, scaled by a weight *c*_*i*_, and some external (e.g., vestibular) input, *h*_*i*_, so that

(3) $$r_{R,i}={\left[a_{i}X_{R}-c_{i}X_{L}+h_{i}\right]}_{+}$$

(4) $$r_{L,i}={\left[a_{i}X_{L}-c_{i}X_{R}+h_{i}\right]}_{+}$$

where *i* = 1, …, *n* indexes the neuron, and [·]_+_ is a threshold-linear function (**Figure A1A**). For simplicity, we assume complete symmetry of the two systems, so that neurons in the two populations have exactly the same set of parameters values.

#### Tuning curve constraints

Using the (abstract) threshold-linear tuning curves, we can describe the firing rates of the right and left position neurons, *r*_*R,i*_ and *r*_*L,i*_, as a function of the eye position *θ* (see Figure 1C), so that (for *i* = 1, …, *n*)

(5) $$r_{R,i}={\left[s_{i}\left(\theta -t_{i}\right)\right]}_{+}$$

(6) $$r_{L,i}={\left[-s_{i}\left(\theta -t_{i}\right)\right]}_{+}$$

where *s*_*i*_ and *t*_*i*_ are the tuning curves' slope and threshold, which are assumed to obey the recruitment order (Aksay et al., 2000) (see **Figure A2**). Since the parameters *s*_*i*_ and *t*_*i*_ are given by the data, they constrain the possible choices of *a*_*i*_, *c*_*i*_, *h*_*i*_ from Equations (3,4). More specifically, on the curve defined by Equation (1), the following relations need to hold:

(7) $$s_{i}=\frac{a_{i}+c_{i}}{2}$$

(8) $$t_{i}=-\frac{\left(a_{i}-c_{i}\right)\beta +2h_{i}}{a_{i}+c_{i}}.$$

To obtain the constraints on the excitatory and inhibitory inputs, *a*_*i*_ and *c*_*i*_, as well as the external inputs, *h*_*i*_, we need to invert this relationship. Since there are three parameters on one side (*a*_*i*_, *c*_*i*_, *h*_*i*_) and two on the other (*s*_*i*_, *t*_*i*_), this inversion is not unique. We therefore introduce a free parameter λ_*i*_ to obtain

(9) $$a_{i}=2\lambda_{i}s_{i}$$

(10) $$c_{i}=2\text{​}\left(1-\lambda_{i}\right)s_{i}$$

(11) $$h_{i}=s_{i}\text{​}\left(1-2\lambda_{i}\right)\beta -s_{i}t_{i}.$$

The free parameter λ_*i*_ ∈ [0, 1] controls the relative role of self-excitation and mutual inhibition. For λ_*i*_ = 0, a neuron receives only inhibitory inputs from the other side, and for λ_*i*_ = 1, it receives only excitatory inputs from the same side. No matter how λ_*i*_ is chosen, as long as *a*_*i*_, *c*_*i*_, and *h*_*i*_ follow the above equations, the measured recruitment order will be obeyed (see **Figure A2**).

#### Stationarity constraints

Next, we need to make sure that the firing rates of the neurons are in a stationary state during fixations of the eye. To do so, we have to define how the individual neural firing rates combine to give rise to the synaptic population activities *X*_*L*_ and *X*_*R*_.

*Assumption 4 (Synaptic Population Activity)*. We assume that the synaptic population activities are generated through a linear combination of the individual neural activities such that (for the right population)

(12) $$X_{R}=\sum_{i=1}^{n}b_{i}g\text{​}\left(r_{R,i}\right)$$

where *b*_*i*_ is a weighting factor, that determines the contribution of the *i*-th neuron to the synaptic population activity and *g*(·) is a sigmoidal function that captures possible non-linearities. These non-linearities can capture saturations in the contribution of individual neural firing rates to the synaptic population activity.

Plugging Equations (2,5) into the above equation, and using the relation *X*_*L*_ = β − *X*_*R*_, we obtain the following condition for stationarity:

(13) $$X_{R}=\sum_{i=1}^{n}b_{i}H\text{​}\left(s_{i}\left(2X_{R}-\beta -t_{i}\right)\right)\text{ with }H\left(\cdot \right)=g\left({\left[\cdot \right]}_{+}\right)$$

Here, *H*(·) is a neural input-output function that combines the effect of the neuron's threshold-linear tuning curves and other non-linearities modeled through the sigmoidal function *g*(·), such as synaptic saturation (see **Figure A1B**). For instance, *H*(·) could be a function similar to the one shown in **Figure A1C**. To obtain a continuum of stationary solutions, i.e., solutions to the above equation for many values of *X*_*R*_, the parameters *b*_*i*_ can be fit so that the above relation holds approximately (see **Figures A1G,H**) (Seung, 1996; Eliasmith, 2005; Machens and Brody, 2008). Since the input-output functions *H*(·) are shifted with respect to the *X*_*R*_-axis due to the differently distributed threshold values *t*_*i*_, a workable solution usually exists. We note that with this fitting, we recover the desired line of synaptic population activities in the normal working regime (see Equation (1) and **Figure A1I**).

#### Input-output functions

Before fitting the parameters *b*_*i*_, we need to make a specific choice for the input-output function *H*(·). Our choice will be driven by a quest for simplicity:

*Assumption 5 (Input-output function)*. We assume that the possible synaptic saturation can be modeled by a heaviside function so that

(14) $$H(x)=\begin{array}{c} 0\text{ if}x\leq 0\\ 1\text{ if}x>0 \end{array}$$

In this case, the summation of a set of Heaviside functions *H*(·) in Equation (13) results in a function that resembles a staircase (see **Figure A1H**). In turn, a particularly simple solution is given if we assume that all *b*_*i*_ = *b* are the same, and that the *t*_*i*_ are equally spaced across the whole range of eye positions. In this case, the staircase approximates a linear function whose slope is determined by *b*, and we only need to set this single parameter to its proper value. This solution assumes, though, that the thresholds *t*_*i*_ extend over the whole eye position range which has not been observed experimentally. Rather, the thresholds cluster in only half the eye position range (as indicated in Figure 1C). However, the framework can easily incorporate this constraint by making the assumption that a) *H*(·) does not saturate completely and is a concave function for large inputs, and b) *H*(·) features high-synaptic thresholds. This has indeed been the assumption in the past (Aksay et al., 2007). In this case, the parameters *b*_*i*_ have to be fit by linear regression. Use of the Heaviside function, however, simplifies the mathematics and makes the underlying architecture more transparent (Machens and Brody, 2008).

#### Dynamics of the network

To develop a dynamical equation for the network and clarify the network connectivity, we define the effective (or synaptic) output of a neuron as

(15) $$x_{R,i}=g\text{​}\left(r_{R,i}\right)$$

so that Equation (12) becomes

(16) $$X_{R}=\sum_{i}b_{i}x_{R,i}.$$

Identical equations hold for the left population. Using Equations (3,4), we can now reformulate the stationarity condition on the level of single cells, using the effective outputs of the neurons,

(17) $$x_{R,i}=H\left(a_{i}X_{R}-c_{i}X_{L}+h_{i}\right)$$

(18) $$=H\left(\sum_{j=1}^{n}a_{i}b_{j}x_{R,j}-\sum_{j=1}^{n}c_{i}b_{j}x_{L,j}+h_{i}\right)\text{​}.$$

Just as the population equations, these equations have solutions for a continuum of values of *x*_*R,i*_ as long as the parameters *b*_*i*_ are fitted as described above. To equip the network with dynamics, we need to assume how the neural activities relax to this stationary state once the system is perturbed. Let us define the following abbreviations,

(19) $$w_{ij,E}=a_{i}b_{j},$$

(20) $$w_{ij,I}=c_{i}b_{j},$$

where *w*_*ij,E*_ denotes the weight of an excitatory connection from neuron *j* to neuron *i* and *w*_*ij,I*_ denotes the weight of an inhibitory connection from neuron *j* to neuron *i*.

*Assumption 6 (Exponential relaxation)*. We assume that the neural activities relax exponentially to the stationary state. This leads to a network with standard Wilson-Cowan dynamics (Dayan and Abbott, 2001),

(21) $$\tau {\dot{x}}_{R,i}(t)=-x_{R,i}(t)+H\left(\sum_{j=1}^{n}w_{ij,E}x_{R,j}-\sum_{j=1}^{n}w_{ij,I}x_{L,j}+h_{i}\right)$$

where now each neuron *i* receives excitatory and inhibitory inputs from the two populations. An identical equation holds for the neurons in the left population and is obtained by switching the *L* and *R* subscripts.

Firing rate dynamics are assumed to be very fast as compared to synaptic dynamics, and are therefore always in equilibrium:

(22) $$r_{R,i}={\left[\sum_{j=1}^{n}w_{ij,E}x_{R,j}-\sum_{j=1}^{n}w_{ij,I}x_{L,j}+h_{i}\right]}_{+}$$

As previously, an identical equation holds for the neurons in the left population and is obtained by switching the *L* and *R* subscripts.

In this model, neurons are excitatory to their own population and inhibitory to the opposing population. However, we can build equivalent models obeying Dale's law, i.e., where neurons are either excitatory or inhibitory, but not both at the same time (unpublished results). A systematic way of mapping networks with mixed excitatory and inhibitory neurons to networks that obey Dale's law was recently proposed in Parisien et al. (2008).

As can be seen in Equations (19,20), we here assume low-rank connectivity. This connectivity has been a standard assumption of all previously proposed models of the oculomotor integrator (Seung, 1996; Aksay et al., 2007) for two reasons: (1) the experimentally observed stable activities across cells in the integrator along the eye position range suggest that the system has low dimensional dynamics, characteristic of a low-rank connectivity system; (2) this assumption simplifies the theoretical treatment of the problem. However, the assumed connectivity should only be viewed as an effective connectivity rather than a direct mapping onto biophysical synapses. Mathematically, one can relax the low-rank assumption: the connectivity matrix can be expanded into “modes” (using singular value decomposition, e.g.), and the dynamics of the oculomotor integrator are simply governed by the first and strongest mode which governs the dynamics in the plane of population activities. However, one could add many more weaker (and orthogonal) modes that would only impact the transient dynamics of single neurons, while leaving the dynamics of the summed population intact. These weaker modes can change the (biophysical) connectivity in many ways, while leaving the effective connectivity the same.

### Theoretical framework: from network activity to eye positions

#### Neural integrator mapping to eye positions

Oculomotor plant dynamics are the result of the innervation of two antagonist muscles (medial rectus and lateral rectus) by motor neurons delivering the position signal (Figure 1A). Consequently, we assume that the eye position θ is related to the difference between right and left motor population activities, making the function *f*_*x*_ between synaptic population activities (*X_R_, X_L_*) and eye position non-linear (see Appendix for details).

#### Oculomotor plant dynamics

To model the observed inertia or sluggishness of the whole system in response to the perturbations (most likely a consequence of muscle inertia and eye dynamics) we implemented a simple exponential decay toward the network stable activities with a time constant τ_θ_, so that

(23) $$\tau_{\theta }\dot{\theta }=-\theta +f_{x}\text{​}\left(X_{R},X_{L}\right).$$

By visual inspection of zebrafish eye traces, we chose τ_θ_ = 200 ms.

### Unilateral inactivation

Now that we have fully determined the relationship between the integrator neural activities and the eye position, we can appropriately interpret the pharmacological inactivation results of (Aksay et al., 2007). After unilateral silencing of the oculomotor integrator, the animals manage to fixate the eyes on the contralateral half of the motor range, and the activities of the intact neurons remain stable within this range. In our framework, this suggests a range of stable points on the upper half of the activity axis *X*_*R*_ and *X*_*L*_. These stable ranges provide an important constraint for the ILA and NP models, limiting the possible choices of λ_*i*_ that we can make in Equations (9–11).

### Parameter tuning of network models

We have clarified the dynamics along the axes required by the pharmacological inactivation results of Aksay et al. (2007), so that we can proceed to the final tuning of the network model.

Independent of the choice of λ_*i*_, the proposed network connectivities feature a line attractor in the (*X_R_, X_L_*) space that agrees with the recruitment order. However, the desired dynamics outside of the attractor, e.g., the dynamics along the axes required by the pharmacological inactivation results, further constrain the tuning of the parameter λ_*i*_. Given the low number of neurons and simplicity of the tuning, we manually tuned this parameter to change the dynamics around the line attractor. However, a least squares optimization procedure could also be built to solve the problem.

In **Figure A4**, we present two model solutions presented in the main section, the independent line attractor model (ILA model) and the null position model (NP model) (see Appendix for the detailed parameter tuning procedure). In general, the nullcline of each half OI is the set of points in space where the dynamics in the corresponding direction are zero. In both ILA and NP models, we tuned the nullclines of either side to consist of densely arranged parallel lines, such that the vectors in the flow field are bound to be either horizontal or vertical. For instance, in the NP model, left range, vertical nullclines of the right side (in blue) ensure that the system has weak dynamics on the horizontal direction, and therefore the dynamics are mostly on the vertical direction.

We note that in the NP model, we have tuned the nullclines to end close to the line of fixed points, to ensure that each piece of nullcline intersects only one piece of the opposite nullcline and therefore obtain a line of fixed points (**Figure A4B**, left). If we relax that assumption and allow for a band attractor for instance, then the nullclines do not have to end as close to the diagonal.

### ILA model dynamics

The ILA model with 36 neurons on each half of the integrator is illustrated in **Figure A4A**. As can be seen, the nullclines resulting from the summation of the input-output functions H(.) of the individual neurons intersect in a line of stable points (**Figure A4A**, left). Furthermore, after complete left inactivation, the system is able to maintain stable positions in the right range (results not shown), as obtained in the pharmacological experiments (Aksay et al., 2007). Finally, this model shows the experimentally observed recruitment order feature, i.e., the higher the threshold the higher the slope of the tuning curves (**Figure A2**), since it was imposed into the model. The dynamics of the ILA model are qualitatively similar to the dynamics of the model developed in Aksay et al. (2007) (see **Figure A4A**, right). The respective network parameters are listed in **Table A1**.

### NP model dynamics

In **Figure A4B**, we illustrate the NP model. Just as the ILA model, the system is able to maintain stable eye positions in half the original range after unilateral silencing. The model differs from the ILA model in one important aspect, however. The upper half of the nullcline (**Figure A4B**, left) is now maintained largely by self-excitation. In fact, the two halves of the oculomotor integrator are almost independent, and the threshold of the neurons is mostly determined by the self-excitatory inputs. However, due to the weak, mutual cross-inhibition, this independence is slightly disturbed, leading to the dynamics shown in **Figure A4B**, right. Altogether, the NP model has qualitatively the same dynamics below the line attractor as the ILA model but different dynamics above the line attractor. However, the NP model dynamics are slower than the ILA model dynamics in the whole state space, given the low inter-dependence of the two areas in the NP model (the arrow lengths in **Figure A4A**, right panel, and **Figure A4B**, right panel, are in different scales). The respective network parameters are listed in **Table A1**.

Despite the connectivity differences between ILA model and NP model, since these models share the same qualitative dynamics below the line attractor, NpHR left stimulation leads to very similar results in both models, with minor differences (results not shown).

Due to the use of Heaviside input-output functions and the resulting staircase character of the nullclines, the network implementation of the NP model features additional fine-scale dynamics that are not illustrated in Figures 7A, **A4B** (see detail of the dynamics in **Figure A4C**). These dynamics do not affect the trajectories in the noise-less case, and disappear when more realistic (e.g., sigmoidal) input-output functions are used for tuning the model (simulations not shown).

### Theoretical framework: instantaneous perturbations

As shown in the results section, we experimentally test the range of models by performing instantaneous perturbations with optogenetic tools: inactivation with halorhodopsin (NpHR) and excitation with channelrhodopsin (ChR2). To model the perturbations, it is crucial to understand the effects of optogenetic tools in the activities of stimulated cells.

It has previously been shown (Arrenberg et al., 2009) that inactivation by NpHR stimulation leads to stronger inactivation of high activity cells than low activity cells, so the inactivation effects are approximately divisive. ChR2 stimulation, on the other hand, causes an increase in activity independent of the original activities (Arrenberg et al., 2009). To incorporate optogenetic stimulations in our dynamical systems model, we take these experimental observations into account.

#### Perturbations with halorhodopsin (NpHR)

Given the divisive nature of the inhibitory perturbations, we model these as multiplicative interactions of the input-output function. Assume that a neuron is perturbed with a brief stimulation of magnitude α_*i*_ (0 < α_*i*_ < 1) and duration *T*. We simulate the synaptic output of such a perturbed neuron (from the left) as

(24) $$\begin{array}{l} \tau {\dot{x}}_{L,i}(t)=-x_{L,i}(t)+\left(1-\alpha_{i}D(t)\right)H\text{​}\left(a_{i}X_{L}(t)-c_{i}X_{R}(t)+h_{i}\right),\\ \;i=1,\ldots ,n \end{array}$$

where *D*(*t*) = *H*(*t* − *t*_0_)*H*(*t*_0_ + *T* − *t*) is a unit perturbation pulse, starting at time *t*_0_, and lasting for *T* seconds. Consequently, at the population level, the system is described by the following population equations during an inactivation of the left half of the integrator:

(25) $$\tau {\dot{X}}_{R}(t)=-X_{R}(t)+\sum_{i=1}^{n}H\text{​}\left(a_{i}X_{R}(t)-c_{i}X_{L}(t)+h_{i}\right)$$

(26) $$\begin{array}{l} \tau {\dot{X}}_{L}(t)=-X_{L}(t)+\sum_{i=1}^{n}\left(1-\alpha_{i}D(t)\right)H\text{​}(a_{i}X_{L}(t)-\\ \;c_{i}X_{R}(t)+h_{i}) \end{array}$$

The parameters α_*i*_ are chosen at random from a Gaussian distribution whose mean μ is equal across neurons, but grows linearly with the stimulation intensity. The standard deviation of the distribution of α_*i*_ was given as σ = μ_min_/2 where μ_min_ indicates the smallest perturbation intensity tested. To ensure that the stimulation strength is always positive, negative α_*i*_ were rectified. Since the mapping of the physical stimulation intensities (in mW) onto the network was unknown, we hand-tuned the mean stimulation effect μ to give results in a reasonable range for the ILA and NP models.

#### Perturbations with channelrhodopsin (ChR2)

Given the assumed additive nature of the excitatory perturbations, we model these as external excitatory inputs into the cells:

(27) $$\tau {\dot{X}}_{R}(t)=-X_{R}(t)+\sum_{i=1}^{n}H\text{​}\left(a_{i}X_{R}(t)-c_{i}X_{L}(t)+h_{i}\right)$$

(28) $$\begin{array}{l} \tau {\dot{X}}_{L}(t)=-X_{L}(t)+\sum_{i=1}^{n}H\text{​}(a_{i}X_{L}(t)-c_{i}X_{R}(t)+h_{i}\\ \;+\alpha_{i}D(t)) \end{array}$$

where *D*(*t*) = *H*(*t* − *t*_0_)*H*(*t*_0_ + *T* − *t*) is once more a unit perturbation pulse, starting at time *t*_0_, and lasting for *T* seconds. The parameters α_*i*_ are chosen from Gaussian distributions, as above, with mean μ and fixed standard deviation, equivalent to one half of the mean of the smallest tested intensity, σ = μ/2. As above, the range of intensities tested was hand-tuned for both models, to fall in the experimentally observed range.

#### Perturbations protocol

To simulate the perturbations, we roughly follow the outline of the experimental perturbations. The model is simulated numerically in MATLAB, using the Euler method. Each trial lasts 1.5 s, and is initialized at one stable eye position, and after 0.5 s, is provided a perturbation of the same duration as in experiments (200 and 100 ms for NpHR and ChR2 trials, respectively). This procedure is repeated for perturbations of different stimulation intensities, and several eye positions across the motor range, and typically 4000 times for each condition (initial eye position, perturbation intensity). The population activities in the network are simulated according to Equation (25) and Equation (26) for the NpHR and Equation (27) and Equation (28) for the ChR2 perturbations. Eye positions are assigned to the resulting population activities according to the mappings outlined in Appendix. Just as for the experimental data, we measure the resulting changes in eye position, Δθ, as a function of the eye position θ just before perturbation. For each perturbation intensity, all neurons are assumed to receive a perturbation magnitude taken from a normal distribution, as explained above.

### Matlab package

A Matlab package in which the ILA model and the NP model are implemented is provided in the online supplementary information.

## Author contributions

Pedro J. Gonçalves and Christian K. Machens designed the model. Pedro J. Gonçalves performed the simulations. Aristides B. Arrenberg and Herwig Baier conceived the experiments. Aristides B. Arrenberg and Bastian Hablitzel performed the experiments. Pedro J. Gonçalves, Aristides B. Arrenberg, Bastian Hablitzel, and Christian K. Machens analyzed the data. Pedro J. Gonçalves, Aristides B. Arrenberg, Herwig Baier, and Christian K. Machens wrote the paper. Pedro J. Gonçalves and Aristides B. Arrenberg contributed equally to this work.

### Conflict of interest statement

The authors declare that the research was conducted in the absence of any commercial or financial relationships that could be construed as a potential conflict of interest.
