# Supplementary material for: Optogenetic perturbations reveal the dynamics of an oculomotor integrator
Source: Front Neural Circuits. 2014 Feb 28;8:10. doi: 10.3389/fncir.2014.00010 (PMC3937552; doi:10.3389/fncir.2014.00010)
Supplement: Supplementary file 1 [file Presentation1.PDF]

APPENDIX

PROCEDURE FOR MODEL TUNING

In **Figure A4**, we present two model solutions presented in the main section, the independent line attractor model (ILA model) and the null position model (NP model). For simplicity, we use the Heaviside input-output functions to build the two model solutions. We note, however, that the results are generalizable to smoother, sigmoidal functions (see **Figure A5**). Accordingly, in (X_R, X_L) space, each neuron (for instance, a right neuron) has an output of 0 below the threshold, and b_i above it, with a threshold defined by the line equation

$$a_i X_R - c_i X_L + h_i = 0 \qquad \text{(A1)}$$

As a consequence, since the right nullcline is a sum over these 2D-Heaviside functions (see Equation 13), the nullcline resembles a staircase in 2D, where the Heaviside threshold lines limit the size of the stairs. In practice, we tune the model by carefully placing the Heaviside threshold lines (A1) in the (X_R, X_L) space. This is done in six steps: the first three steps correspond to the tuning of the dynamics, and the last three steps correspond to the computation of the network parameters that ensure the dynamics. For example, for the right neurons:

1. Choose a concrete value for β. Since our network has 36 neurons, we choose $\beta = 36$.
2. Choose b_i. For simplicity, we assume $b_i = 1$.
3. Design the nullcline of the right population activity, and desired right population activity dynamics outside of the attractor. The nullcline is defined as the stationary solutions X_R to the equation

$$X_R = \sum_{i=1}^{n} b_i H(a_i X_R - c_i X_L + h_i) \qquad \text{(A2)}$$

   The nullcline resembles a staircase in 2D, where each neuron introduces a stair. To design the nullcline, we carefully choose the lower and upper limits $(X_{Li}^{\text{low}}, X_{Li}^{up})$ of each stair at $X_{Ri}^{*} = i\ (i = 0, \ldots, n)$.
4. In turn, these limits correspond to points on the Heaviside threshold lines (see Equation A1), and allow us to compute the respective line equations. For example, in the NP model, the stair defined at $X_{R4} = 4$ has the lower limit $X_{L4}^{\text{low}} = 31.5$, and the stair at $X_{R5} = 5$ has the upper limit $X_{L5}^{up} = 49$. Therefore, we have two points with which we can compute the slope and y-intercept of the threshold line for neuron 5:

$$\text{slope} = X_{L5}^{up} - X_{L4}^{\text{low}} = 17.5 \qquad \text{(A3)}$$

$$\text{y-intercept} = X_{L5}^{up} - \text{slope} X_{R5} = -38.5 \qquad \text{(A4)}$$

   Given the Heaviside threshold line equations (A1) and the relation between λ and the parameters a_i and c_i (see Equations 9,10), the 'slope' and 'threshold' allow us to compute the parameters λ_i. We note that with this procedure, the tuning curve thresholds t_i are approximately equally spaced across the whole range.
5. Choose tuning curves slopes s_i. To ensure the recruitment order feature (**Figure A2**), these slopes should monotonically increase with t_i, i.e., these should increase for neurons crossing the line attractor $X_R + X_L = \beta$ further to the right.
6. After the steps above, we have determined all the parameters $(s_i, t_i, \lambda_i, \beta)$. As such, we can compute the network parameters (a_i, c_i, h_i) following Equations 9–11.

We assume the two halves of the integrator are symmetric so that left neurons have the same parameters as right neurons.

The ILA and NP model nullclines and dynamics are illustrated in **Figure A4**. A table with the parameter values for both ILA model and NP model is provided in **Table A1**.

NEURAL INTEGRATOR MAPPING TO EYE POSITION

In our study, we perturbed the oculomotor integrator and thereby observed its dynamics beyond its normal operating regime. Consequently, we need to determine the function between eye position and synaptic population outputs in the whole space (X_R, X_L). We have previously defined how eye position relates to the synaptic population outputs (X_R, X_L) in the normal operating regime (see Equation 2), but we still lack a model of how they relate outside of this regime.

To build a model of the complete mapping, we will start on the motor side. Horizontal eye movements are the result of the innervation of two antagonist muscles (medial rectus and lateral rectus) by motor neurons (**Figure 1A**). The tuning curves of these motor neurons have been determined in the goldfish (Pastor et al., 1991), see **Figure A3A**, so that we can relate the activity of the motor neuron population to the eye position in the system's normal operating. We define the motor population activities as m_R^* and m_L^* and write

$$m_R^* = \sum_{i=1}^{n} r_{mR,i}^*(\theta) = G(\theta) \qquad \text{(A5)}$$

$$m_L^* = \sum_{i=1}^{n} r_{mL,i}^*(\theta) = G(-\theta) \qquad \text{(A6)}$$

where $r_{mR,i}^*(\theta)$ and $r_{\text{mL},i}^*(\theta)$ are the activities of individual right and left motor neurons (see **Figure A3A**), and θ is the eye position as before. The star indicates that the relation holds only in the system's normal operating regime. The (non-linear) dependency of the motor population activities as a function of eye position is indicated by the function $G(\cdot)$ (see dashed lines in **Figure A3B**). Note that for simplicity we assume that there are as many motor neurons as position neurons, i.e., n motor neurons on each side.

Next, we want to invert and generalize this relation, i.e., decide how the eye positions depends on arbitrary combinations of the left and right motor population activities. For simplicity, we will assume that the eye position is determined by the difference between m_R and m_L. When the system is operating normally, the difference of population activity maps onto eye position as

$$\theta = f_m\left(m_R^* - m_L^*\right) \qquad \text{(A7)}$$

Table A1 | Network parameter values for ILA model and NP model: position neurons.

	ILA model			NP model		
**neuron\parameter**	**a**	**c**	**h**	**a**	**c**	**h**
**1**	0.00	0.20	7.10	0.19	0.011	0.38
**2**	0.00	0.60	20.70	0.57	0.03	0.55
**3**	0.00	1.00	33.50	0.95	0.05	-0.08
**4**	0.00	1.40	45.50	1.32	0.08	-1.51
**5**	0.00	1.80	56.70	1.70	0.10	-3.75
**6**	0.00	2.20	67.10	2.08	0.12	-6.78
**7**	0.00	2.60	76.70	2.46	0.14	-10.61
**8**	0.00	3.00	85.50	2.84	0.16	-15.24
**9**	0.00	3.40	93.50	3.22	0.18	-20.68
**10**	0.00	3.80	100.70	3.59	0.21	-26.91
**11**	0.00	4.20	107.10	3.97	0.23	-33.94
**12**	0.00	4.60	112.70	4.35	0.25	-41.77
**13**	0.00	5.00	117.50	4.73	0.27	-50.41
**14**	0.00	5.40	121.50	5.11	0.29	-59.84
**15**	0.00	5.80	124.70	5.49	0.31	-70.07
**16**	0.00	6.20	127.10	5.86	0.34	-81.10
**17**	0.00	6.60	128.70	6.24	0.36	-92.94
**18**	0.00	7.00	129.50	6.62	0.38	-105.57
**19**	7.23	0.17	-131.13	7.26	0.14	-135.64
**20**	7.61	0.19	-145.65	7.66	0.14	-150.78
**21**	7.99	0.21	-160.90	8.05	0.15	-166.71
**22**	8.38	0.22	-176.88	8.44	0.16	-183.44
**23**	8.76	0.24	-193.59	8.83	0.17	-200.97
**24**	9.13	0.27	-211.03	9.23	0.17	-219.30
**25**	9.51	0.29	-229.20	9.62	0.18	-238.44
**26**	9.89	0.31	-248.10	10.01	0.19	-258.37
**27**	10.26	0.34	-267.72	10.41	0.19	-279.10
**28**	10.64	0.36	-288.08	10.80	0.20	-300.63
**29**	11.01	0.39	-309.17	11.19	0.21	-322.97
**30**	11.38	0.42	-331.00	11.58	0.22	-346.10
**31**	11.75	0.45	-353.56	11.98	0.22	-370.03
**32**	12.12	0.48	-376.86	12.37	0.23	-394.76
**33**	12.49	0.51	-400.88	12.76	0.24	-420.29
**34**	12.85	0.55	-425.61	13.15	0.25	-446.63
**35**	13.22	0.58	-451.05	13.55	0.25	-473.76
**36**	13.58	0.62	-477.16	13.94	0.26	-501.69

For each model (ILA and NP model), parameter values are shown for each neuron on one population (left or right OI). We assume complete symmetry of the two populations, so that neurons in the two populations have exactly the same set of parameters values.

Table A2 | Network parameter values for ILA model and NP model: motor neurons.

	ILA model			NP model		
**neuron\parameter**	**d**	**e**	**k**	**d**	**e**	**k**
**1**	0.00	0.50	-179.94	0.47	0.03	-167.16
**2**	0.00	1.50	-166.34	1.42	0.08	-167.00
**3**	0.00	2.50	-153.54	2.36	0.14	-167.63
**4**	0.00	3.50	-141.54	3.31	0.19	-169.06
**5**	0.00	4.50	-130.34	4.26	0.24	-171.29
**6**	0.00	5.50	-119.94	5.20	0.30	-174.33
**7**	0.00	6.50	-110.34	6.15	0.35	-178.16
**8**	0.00	7.50	-101.54	7.09	0.41	-182.79
**9**	0.00	8.50	-93.54	8.04	0.46	-188.22
**10**	0.00	9.50	-86.34	8.99	0.51	-194.46
**11**	0.00	10.50	-79.94	9.93	0.57	-201.49
**12**	0.00	11.50	-74.34	10.88	0.62	-209.32
**13**	0.00	12.50	-69.54	11.82	0.68	-217.95
**14**	0.00	13.50	-65.54	12.77	0.73	-227.39
**15**	0.00	14.50	-62.34	13.72	0.78	-237.62
**16**	0.00	15.50	-59.94	14.66	0.84	-248.65
**17**	0.00	16.50	-58.34	15.61	0.89	-260.48
**18**	0.00	17.50	-57.54	16.55	0.95	-273.12
**19**	18.07	0.43	-318.17	18.16	0.34	-303.19
**20**	19.03	0.47	-332.69	19.14	0.36	-318.32
**21**	19.99	0.51	-347.94	20.12	0.38	-334.26
**22**	20.94	0.56	-363.92	21.11	0.39	-350.99
**23**	21.89	0.61	-380.63	22.09	0.41	-368.52
**24**	22.84	0.66	-398.07	23.07	0.43	-386.85
**25**	23.78	0.72	-416.24	24.05	0.45	-405.98
**26**	24.72	0.78	-435.14	25.03	0.47	-425.92
**27**	25.66	0.84	-454.76	26.01	0.49	-446.65
**28**	26.59	0.91	-475.12	27.00	0.50	-468.18
**29**	27.53	0.98	-496.21	27.98	0.52	-490.51
**30**	28.45	1.05	-518.04	28.96	0.54	-513.65
**31**	29.38	1.12	-540.60	29.94	0.56	-537.58
**32**	30.30	1.20	-563.90	30.92	0.58	-562.31
**33**	31.22	1.28	-587.92	31.90	0.60	-587.84
**34**	32.14	1.37	-612.65	32.89	0.61	-614.17
**35**	33.05	1.46	-638.09	33.87	0.63	-641.31
**36**	33.95	1.55	-664.20	34.85	0.65	-669.24

For each model (ILA and NP model), parameter values are shown for each neuron on one population (left or right motor neuron population). We assume complete symmetry of the two populations, so that neurons in the two populations have exactly the same set of parameters values.

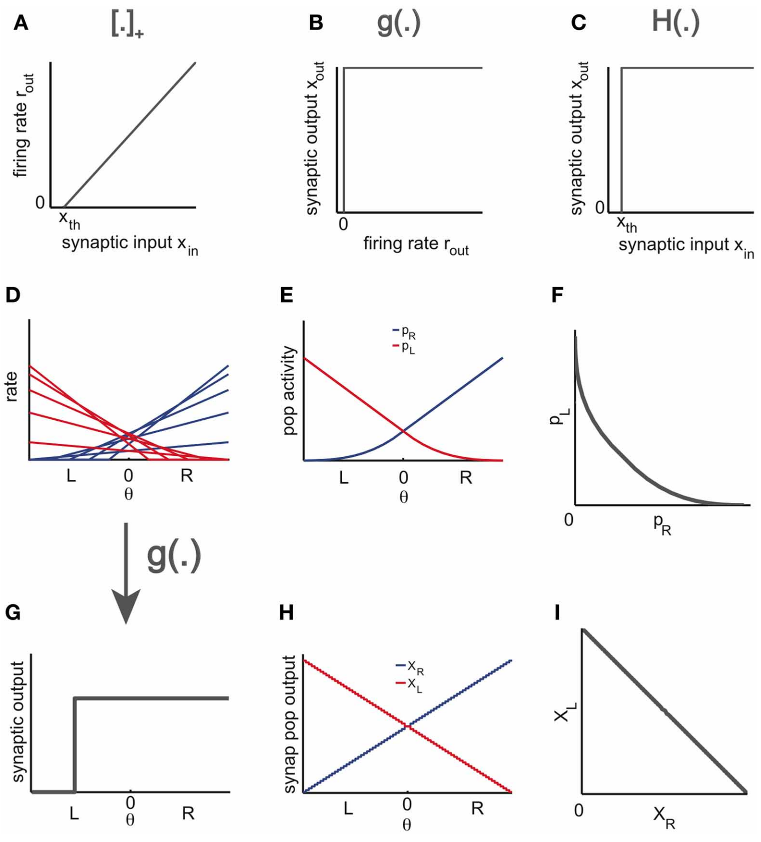

FIGURE A1 | Single neuron model and population activity space. **(A–C)** Single neuron model (Machens and Brody, 2008). **(A)** Threshold linear mapping $[\cdot]$ from synaptic input x_{in} to firing rate output r_{out}. The neuron has a threshold x_{th} which the input has to pass to generate a firing response. **(B)** Saturating mapping $g(\cdot)$ from the neuron's firing rate r_{out} to synaptic output x_{out}. **(C)** The synaptic output x_{out} is related to the synaptic input x_{in} by the function $H(\cdot)$. x_{th} has the same definition as in **(A)**. **(D)** Abstracted tuning curves of right (blue) and left (red) position cells, based on (Aksay et al., 2007). **(E)** Population activity of right and left half of the integrator as a function of eye position. **(F)** Line of population activities in the normal operating regime. **(G)** Single-cell synaptic activity curve of a right position cell. **(H)** Fitted synaptic population activities of right and left half of the integrator as a function of eye position. **(I)** From the fitted synaptic population activities in **(H)**, we obtain the desired line of synaptic population activities in the normal working regime.

where $f_m(\cdot)$ is a non-linearity determined by the data. It can be read off from **Figure A3C**. Note that, as before, right range positions are defined as positive eye positions, and left range positions as negative. As can be seen in **Figure A3C**, the stable eye positions are approximately equal to the difference between the motor population activities, m_R^* and m_L^*. We are going to assume that this function generalizes to the whole motor population space (m_R, m_L), so that $\theta = f_m(m_R - m_L)$.

In the next step, we need to determine the relation between position neuron activities and motor neuron activities. Again, we will first focus on the system's normal operating regime and relate the tuning curves of position and motor neurons. From (Pastor et al., 1991; Aksay et al., 2000), we can see that in the goldfish, motor neuron tuning curves have on average higher slopes (2.5 times) and more central thresholds (shift of around 15 degrees toward the center) than position neuron tuning curves (compare **Figure 1C** and **Figure A3A**). Therefore, the sums of the right and left motor tuning curves, m_R^* and m_L^* respectively, are non-linearly related to the sums of the right and left position neuron tuning curves, p_R^* and p_L^* respectively,

$$m_R^* = f\left(p_R^*\right) \tag{A8}$$

$$m_L^* = f\left(p_L^*\right). \tag{A9}$$

where p_R^* and p_L^* are defined as the sums of the individual firing rates $r_{R,i}$ and $r_{L,i}$ (**Figures 1C,D**), respectively:

$$p_R^* = \sum_{i=1}^{n} r_{R,i} \tag{A10}$$

$$p_L^* = \sum_{i=1}^{n} r_{L,i} \tag{A11}$$

The relation (A8), and thereby the function $f(\cdot)$, is plotted in **Figure A3B** (inset). This function is determined by the data obtained while the system is working normally; for simplicity, we will assume that the function generalizes to the whole activity space, i.e., for (p_R, p_L) to (m_R, m_L). As a result, m_R changes

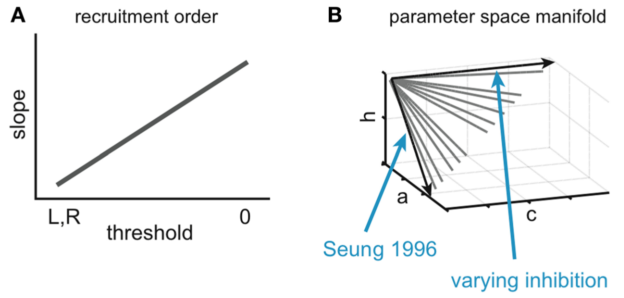

FIGURE A2 | Recruitment order. (A) Recruitment order of position cells: the more central the tuning curve threshold, the bigger the slope of the tuning curve (see tuning curves in **Figure A1D**). **(B)** There is a manifold of solutions to the recruitment order in the parameter space. The parameters *a*, *c*, and *h* can be interpreted as auto-excitation, cross-inhibition and tonic input, respectively. Two specific solutions are highlighted: Seung 1996 model without cross-inhibition (Seung, 1996), and a model exclusively with cross-inhibition.

very little when p_R is small, so that eye position is almost independent of p_R and depends mostly on p_L in the left motor range. The reverse is true in the right motor range.

Finally, we recapitulate the dependencies of the position neuron population activities (p_R, p_L) and their synaptic counter parts, (X_R, X_L). These variables are related supra-linearly (see **Figures 1D,F**),

$$p_R = \sum_{i=1}^{n} r_{R,i} = \sum_{i=1}^{n} [a_i X_R - c_i X_L + h_i]_+ = k(X_R, X_L) \quad \text{(A12)}$$

$$p_L = \sum_{i=1}^{n} r_{L,i} = \sum_{i=1}^{n} [a_i X_L - c_i X_R + h_i]_+ = k(X_L, X_R), \quad \text{(A13)}$$

where the function $k(\cdot, \cdot)$ captures the relevant mapping.

In summary, θ is related to the synaptic population activities, X_R and X_L, as

$$\begin{aligned} \theta &= f_m(m_R - m_L) \\ &= f_m\big(f(p_R) - f(p_L)\big) =: f_p\big(p_R, p_L\big) \\ &= f_p(k(X_R, X_L),\ k(X_L, X_R)) =: f_x(X_R, X_L)\,. \end{aligned} \quad \text{(A14)}$$

The mapping of the synaptic population activities onto eye position, $f_x(X_R, X_L)$, is shown in **Figure A3D**. As a result of (1) the convex relation between (m_R, m_L) and (p_R, p_L), and (2) the supra-linear relation between (p_R, p_L) and (X_R, X_L), the eye position isolines have strong curvatures in the (X_R, X_L)-space (**Figure A3D**).

MOTOR NEURONS PARAMETERS

Given previous findings in the goldfish (Pastor et al., 1991; Aksay et al., 2000), we assume that the motor neurons receive an excitatory input from the ipsilateral integrator population weighted by d_i, an inhibitory input from the opposing integrator population weighted by e_i and and external input k_i, so that the firing rates of motor neurons are given by

$$r_{\mathrm{mR,i}} = [d_i X_R - e_i X_L + k_i]_+ \quad \text{(A15)}$$

$$r_{\mathrm{mL,i}} = [d_i X_L - e_i X_R + k_i]_+ \quad \text{(A16)}$$

where $i = 1, \ldots, n$ indexes the neuron. As with the position neurons, for simplicity we assume that neurons in the two sides have equal parameters values. The parameters of motor neurons for both the ILA and NP models are provided in **Table A2**.

FITTING THE DYNAMICS WITH SMOOTH SIGMOIDS

Discontinuities in the vector field

Since the input-output functions are step-like functions, the vector field shows small discontinuities on the dynamics. However, even if derivatives in a dynamical system are discontinuous, the actual state space trajectory will still be continuous. In our modeling framework, these discontinuities are of two types, and lead to absolutely no theoretical or practical problems:

- Discontinuities where the dynamics switches direction in the transition through the threshold lines of Heaviside functions. These will appear in areas of unstable points. In these cases, smoother input-output functions lead to smooth transitions in the derivatives across the unstable points, and the diverging dynamics around the attractor are qualitatively similar to the case with heaviside functions.
- Discontinuities where the dynamics keep the same direction in the transition through the threshold lines of the Heaviside functions. In these cases, each discontinuity has a magnitude of one Heaviside function, which is much smaller than the overall population activities, therefore having negligible impact on the dynamics. Again, we can relax the Heaviside functions to smooth sigmoidals without any impact on the dynamics of the system.

These discontinuities, which can be seen on a much finer scale, do not affect any of our conclusions: they can be eliminated by moving toward smooth, sigmoidal functions. To illustrate this, we fitted the ILA and NP model dynamics with smoother sigmoidals (please see **Figure A5**). As can be seen, the relaxation of the Heaviside assumption does not qualitatively change our results.

Smooth sigmoids fitting

In this section, we illustrate a fitting procedure of the ILA and NP dynamics with smooth sigmoidal functions.

The original model dynamics are described by:

$$\tau \dot{X}_R(t) = -X_R(t) + \sum_{i=1}^{n} b_i H(a_i X_R(t) - c_i X_L(t) + h_i) \quad \text{(A17)}$$

$$\tau \dot{X}_L(t) = -X_L(t) + \sum_{i=1}^{n} b_i H(a_i X_L(t) - c_i X_R(t) + h_i) \quad \text{(A18)}$$

where $n = 36$, $b_i = 1$ for all i (in the ILA and NP models).

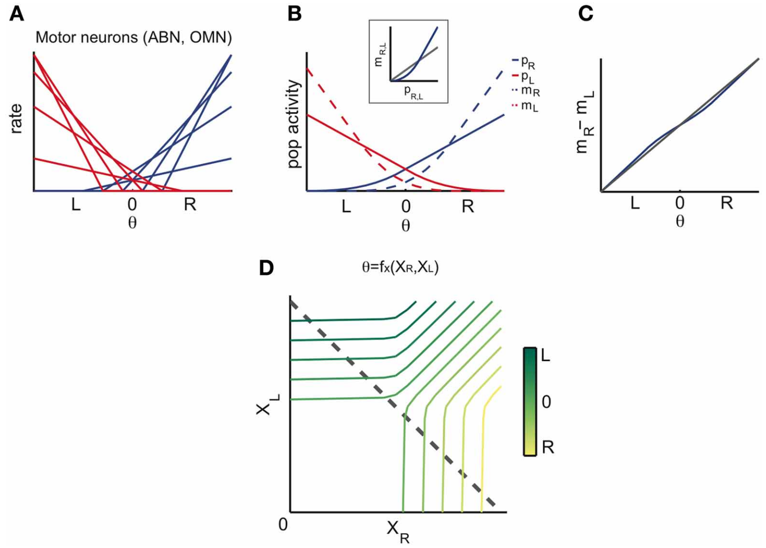

FIGURE A3 | Eye position mapping. (A) Abstracted motor neurons' tuning curves (Pastor et al., 1991). Typically, the thresholds are shifted toward the center and slopes are bigger, when compared to the position neuron tuning curves (compare **Figure 1C**). **(B)** Population activity of right and left half of the integrator (solid lines) and right and left motor neurons (dashed line) as a function of eye position. Inset: the function between motor population activity and ipsilateral position population activity (in blue) is sub-linear for small position population activity, and supra-linear for large position population activity, as evidenced by the identity line in gray. **(C)** Horizontal eye movements are the result of the innervation of two antagonist muscles (medial rectus and lateral rectus) by motor neurons delivering the position signal. Eye position θ is approximately equal to the difference between right and left motor population activities, m_R and m_L. **(D)** Mapping f_x of position synaptic population activities onto eye position is non-linear. The colored iso-lines correspond to different eye positions θ in the (X_R, X_L) space. The gray line indicates the line attractor and thereby the stable equilibrium states of the system.

We fit the dynamics above with the following model:

$$\tau\dot{X}_R(t) = -X_R(t) + \sum_{i=1}^{m} \beta_i g(\alpha_i X_R(t) - \gamma_i X_L(t) + \delta_i) \quad \text{(A19)}$$

$$\tau\dot{X}_L(t) = -X_L(t) + \sum_{i=1}^{m} \beta_i g(\alpha_i X_L(t) - \gamma_i X_R(t) + \delta_i) \quad \text{(A20)}$$

where $m >> n$, $(\alpha_i, \gamma_i, \delta_i)$ are parameters randomly set to span the whole range of possible values of (a_i, c_i, h_i). $g(\cdot)$ is a sigmoidal function given by

$$g(x) = \frac{[x]_+}{10\text{Hz} + [x]_+} \quad \text{(A21)}$$

Given this model, we follow a linear least squares procedure to fit the parameters β_i (constrained to be zero or positive) which make the model Equations (A19,A20) best approximate the dynamics given by Equations (A17,A18). With this procedure, only a subset of the parameters β_i (on the order of the original number of neurons n) is different from zero.

In **Figures A5A,C**, we can see the fitted dynamics of the ILA and NP models, respectively. As can be seen, the dynamics of the original models (**Figures A4A,B**) are well fitted by the corresponding approximations (**Figures A5A,C**), and this by using smooth sigmoids (**Figures A5B,D**).

DATA INCLUDING HIGH INTENSITY STIMULATIONS

We are interested in inferring the dynamics of the system outside of the normal operating regime. In the main section of the paper, we have succeeded to extract the dynamics in the neighborhood of the normal operating regime, by stimulating the system with brief and small intensity stimulations. When we increase the stimulation intensities, the system behaves differently than expected, which could indicate e.g., limitations in the spatial precision of stimulation, or new features in the system's network structure.

More specifically, when we stimulate the left half of the integrator of the NpHR animals with higher intensities (light power above 0.8 mW), new features arise in the right range, i.e., a small eye movement toward the left emerges, and tends to be stronger for higher stimulation intensities and eccentric initial eye positions (**Figures A6A,B**). This slight deviation from the model could be caused by light scattering to the right half of the integrator when stimulating the left half (simulation data not shown). We note a difference between the response of the two eyes, a property that our model does not address: in the left range, the left eye is slightly more responsive than the right eye; in the right range, the right eye is slightly more responsive than the left eye (**Figures A6A,B**). This result might be related to the fact that each integrator half encodes mostly the position of the ipsilateral eye, in agreement with the findings of (Debowy and Baker, 2011). To a first approximation, however, the predictions from our model remain fairly acceptable, accounting for most of NpHR stimulation results.

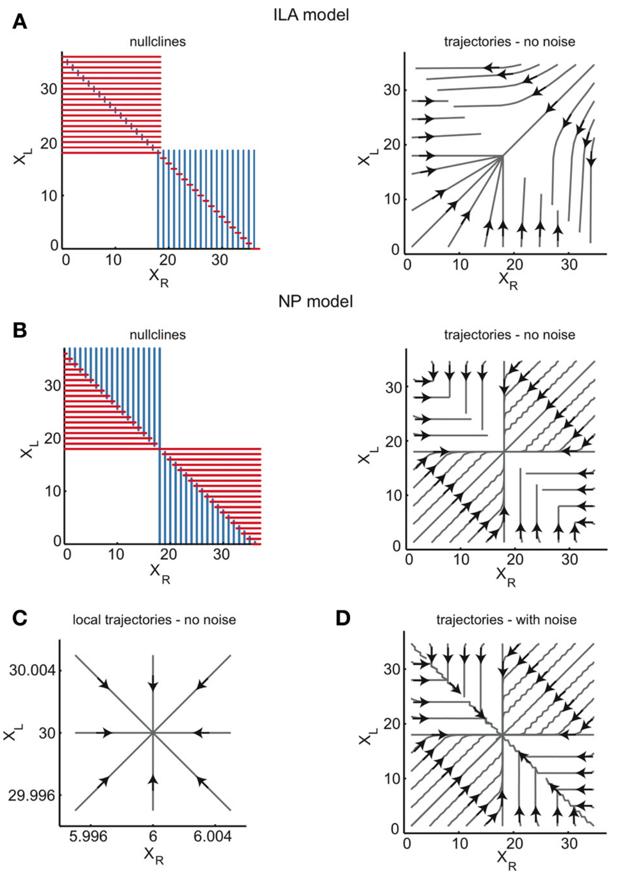

FIGURE A4 | The ILA and NP models: nullclines and dynamics. (A) ILA model and **(B)** NP model. Left: in the (X_R, X_L) space, the blue and red thick lines correspond to the right nullcline and left nullcline respectively. Fixed points are at the intersection of the nullclines. Right: different initial points out of the attractor in the (X_R, X_L) space relax toward the stable points in the line $X_R + X_L = 36$. The corresponding trajectories are in gray, and the black arrows indicate the direction of the flow. **(C)** Fine-scale dynamics in the vicinity of a stable point in the NP model. In all models, the line attractor is approximated through a series of stable fixed points. Here one of these fixed points is shown, including eight different trajectories, relaxing from different initial points onto the fixed point $(X_L, X_R) = (6, 30)$. **(D)** Trajectories of the NP model in the presence of small noise. Same representation as in **(B)**. Noise in the dynamics introduces a small drift toward the central point.

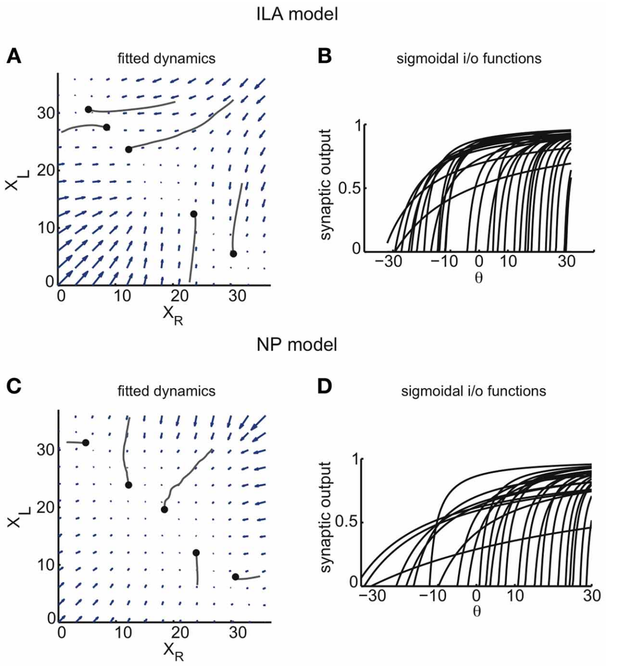

FIGURE A5 | Fitting the dynamics with smooth sigmoids. (A) Fitted ILA model. Vector field of X_R vs X_L, having fixed points approximately in the line $X_R + X_L = 36$. Five different initial points out of the attractor in the (X_R, X_L) space relax toward the stable points indicated by black dots. The corresponding trajectories are in gray. The fitted model has 32 neurons. **(B)** Sigmoidal input-output functions used for the ILA dynamics fit. **(C)** Fitted NP model dynamics. Five different initial points out of the attractor in the (X_R, X_L) space relax toward the stable points indicated by black dots. The corresponding trajectories are in gray. The fitted model has 30 neurons. **(D)** Sigmoidal input-output functions used for the NP dynamics fit.

In ChR2 animals, higher intensity stimulations (light power above 0.8 mW) induce movements toward the right in the left starting positions, which contrasts with both ILA and NP model predictions (**Figures 3H**, **7H**). However, the results may be explained in the NP model if we assume either that higher intensity stimulations residually affect the opposite half of the integrator, or other parts of the system, such as vestibular neurons. In **Figure 8**, we show how the stimulation of afferents to the integrator, such as vestibular neurons, could account for the ChR2 high intensity stimulations results.

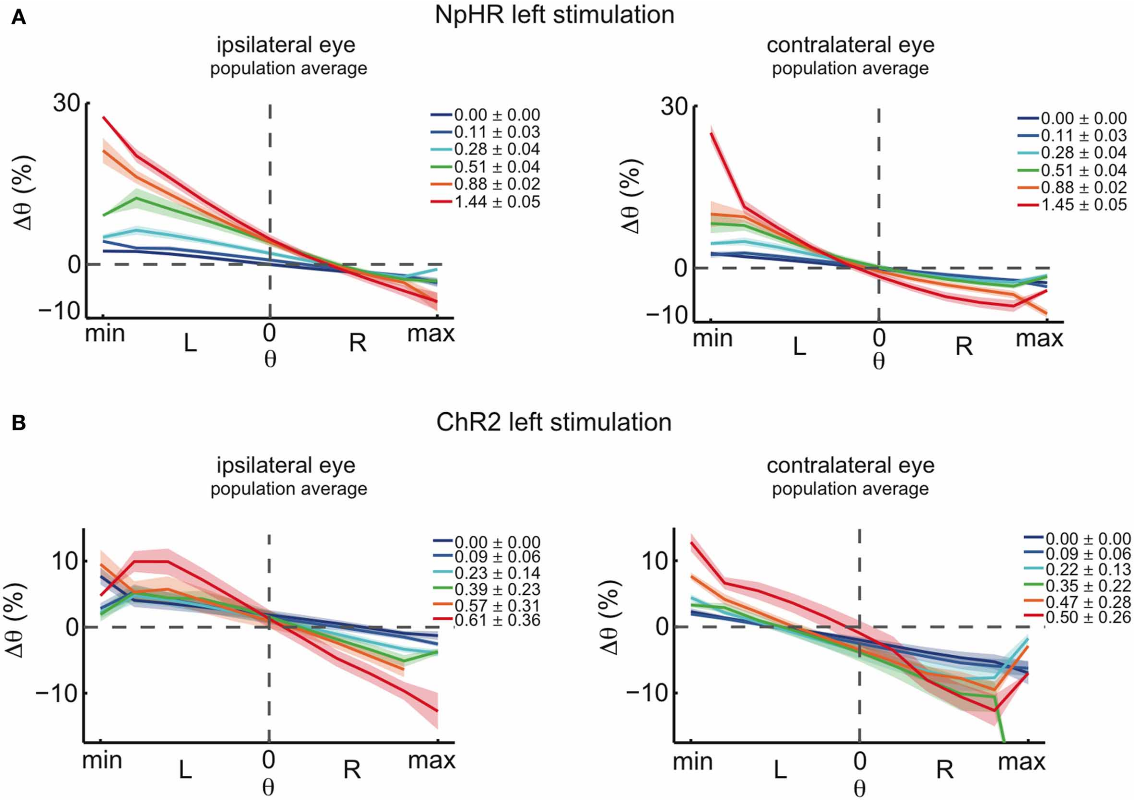

FIGURE A6 | NpHR and ChR2 data including high stimulation intensities and resolved for individual eyes. (A) Eye position changes Δθ caused by NpHR stimulation versus eye position previous to stimulation. Spline fit averages across all tested fish ($n = 24$ recordings). Legends indicate the light power averages (in mW) for each bin and respective standard errors across recordings. In the left range, left eye (left) is more responsive than the right eye (right). In the right range, the right eye is more responsive than the left eye. **(B)** Eye position changes Δθ caused by ChR2 stimulation versus eye position previous to stimulation. Spline fit averages across all tested fish ($n = 19$ recordings). The 2 highest intensity bins generate centripetal movements from both sides.

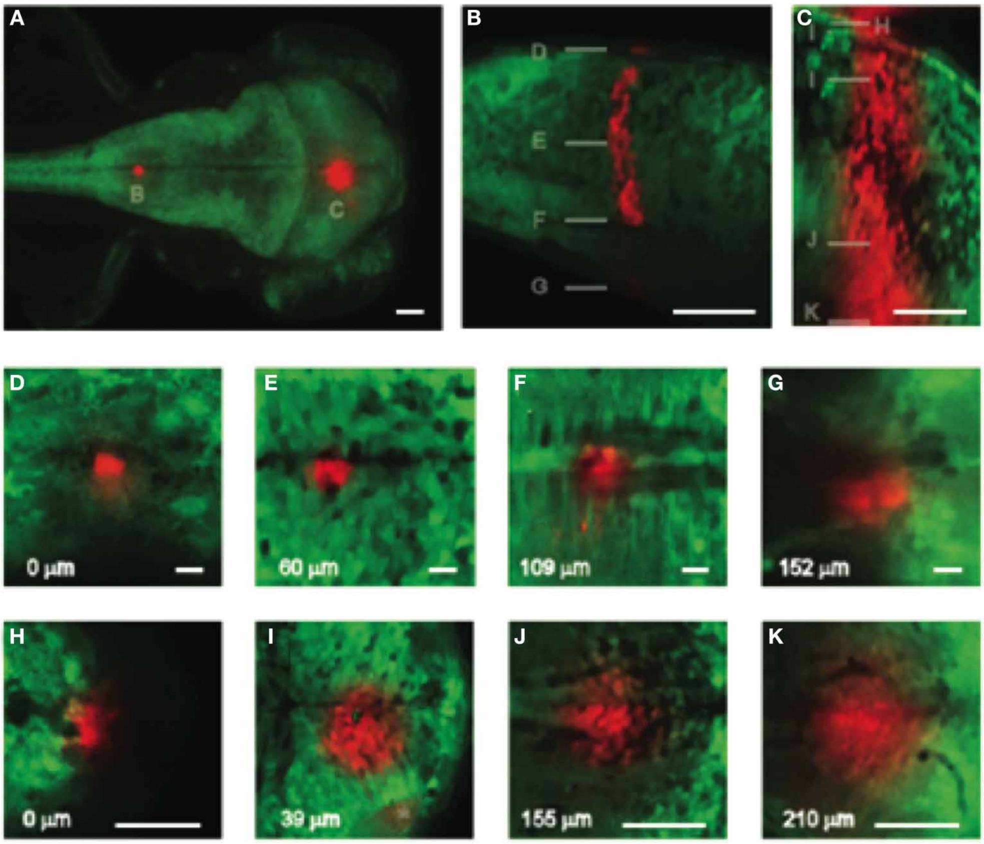

FIGURE A7 | Photoconversion experiments reveal fiber optic light spread within the larval zebrafish. (A) Dorsal view of a zebrafish expressing the photoconvertible fluorophore Kaede [*Et(E1b:Gal4)s1101t, Tg(UAS:Kaede)s1999t*]. In **(B)** a thin optic fiber (10 µm diameter, *NA* = 0.1, Thorlabs HPSC10) has been placed above the hindbrain and Kaede has been photoconverted to red using a UV laser. In **(C)** a 50 µm diameter fiber (*NA* = 0.22) has been placed above the midbrain. **(B)** The photoconverted column imaged from the side. Note the unexpected curvature of the light path which was presumably caused by cell migration during the ~3 h between photoconversion and imaging. **(C)** Side view reconstructed from a dorsal z-stack. **(D–G)** Single optical slices at the levels indicated in **(B)** reveal the lateral spread of light using a 10 µm diameter fiber. **(H–K)** Single optical slices at the levels indicated in **(C)** reveal the lateral spread of light using a 50 µm diameter fiber. Scale bars are 50 µm in **(A–C)** and **(H–K)** and 10 µm in **(D–G)**.

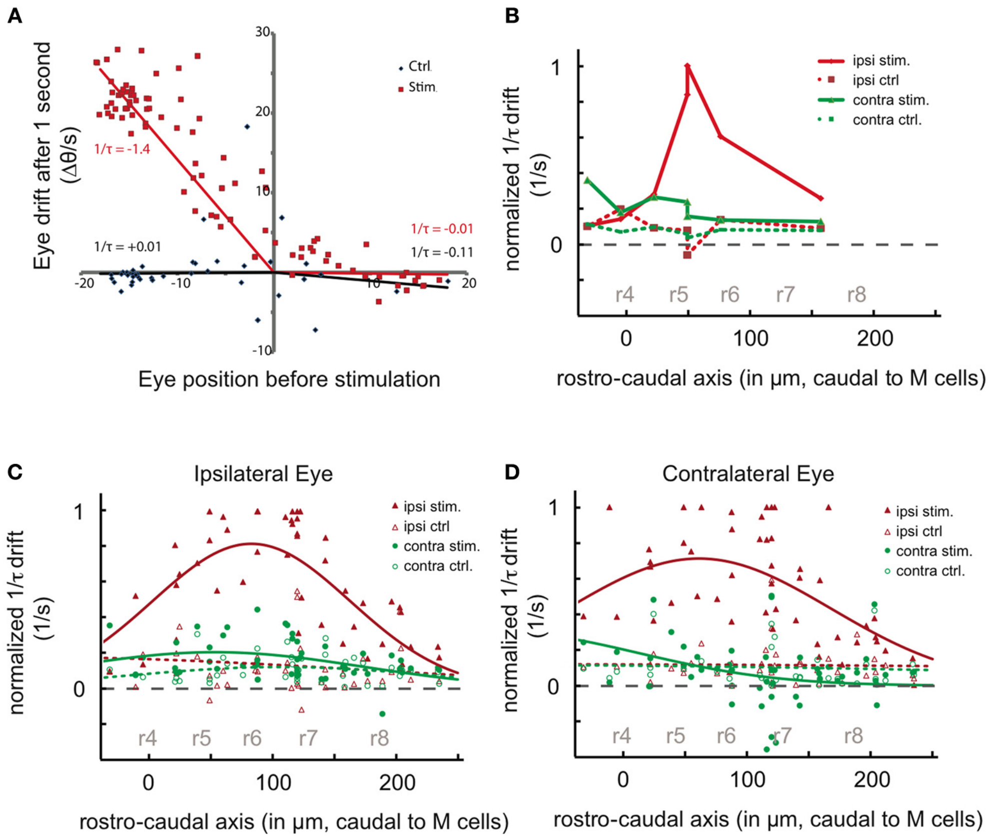

FIGURE A8 | Mapping of NpHR-induced eye drift magnitudes. (A) For each fish, eye and hindbrain position, the eye drift velocity was plotted against the initial eye position. For each eye position range (ipsiversive, contraversive) and stimulation condition (stimulated, control), linear regressions were performed through the origin. The slope of the fit corresponds to the inverse of the eye drift time constant $(1/\tau)$. **(B)** The induced eye drifts of the eye ipsilateral to the stimulation in one fish. **(C,D)** Mapping of eye drift magnitudes. Eye drift magnitudes for the eyes ipsilateral to the stimulation **(C)** and contralateral to the stimulation **(D)** of 7 animals are plotted and Gaussian functions are fitted.

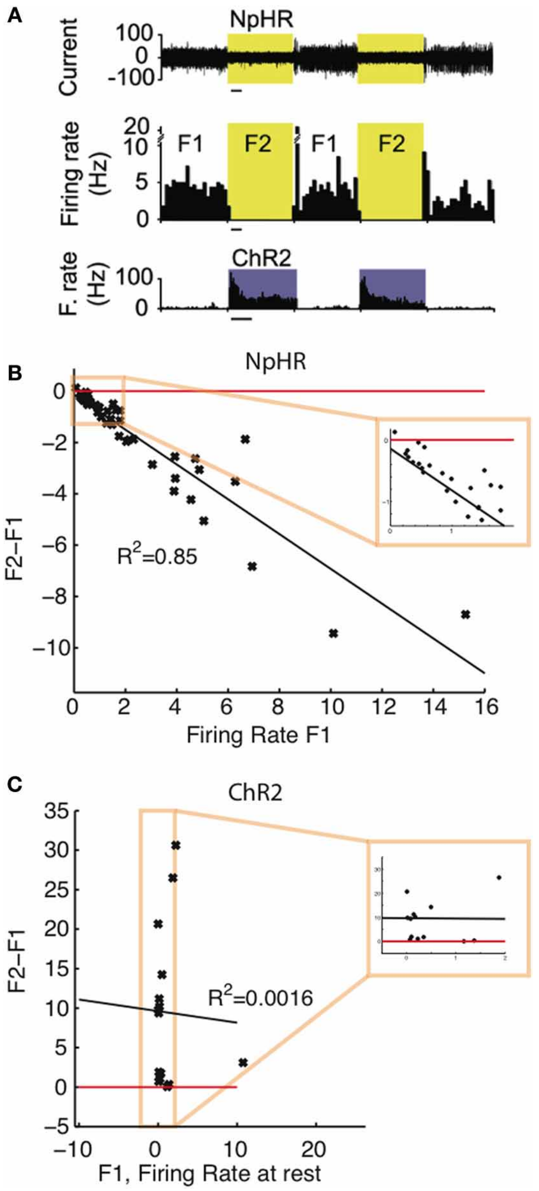

FIGURE A9 | Dependence of optogenetic firing rate manipulation on initial firing rate. (A) The spontaneous firing rate of NpHR or ChR2 expressing neurons in the hindbrain was recorded during light stimulation (F2) and at rest (F1). Unprocessed current trace (top) and corresponding single-unit firing rate histogram (middle) of a NpHR expressing cell that was silenced during illumination (yellow shaded boxes). Bottom: A ChR2 expressing cell fired at a higher rate upon stimulation with blue light (blue shaded boxes). **(A)** Is reproduced from Arrenberg et al. (2009) and the electrophysiological data from this publication was analyzed to generate **(B,C)**. Scale bars: 10 s. **(B)** The difference between the firing rate during illumination and the firing rate at rest (F2-F1) is plotted against the firing rate at rest (F1) for NpHR expressing cells. The firing rate change is dependent on the initial firing rate, as suggested by the linear fit (black line, $R^2 = 0.85$). The inset in the upper right shows a magnified view of the data close to the origin. **(C)** The difference between the firing rate during illumination and the firing rate at rest (F2-F1) is plotted against the firing rate at rest (F1) for ChR2 expressing cells. The firing rate change is independent of the firing rate at rest ($R^2 = 0.0016$). The inset in the upper right shows a

(Continued)

FIGURE A9 | Continued

magnified view of the data close to the origin. Note that the firing rates at rest apparently differ for NpHR and ChR2 expressing cells. This is possibly the result of a biased sampling by the experimenter: for ChR2 expressing cells, many cells that showed almost no firing at rest were selected for ChR2 recordings, whereas for NpHR such cells with low activity were not selected for recordings and instead cells with a higher baseline activity were searched.

REFERENCES

Aksay, E., Baker, R., Seung, H. S., and Tank, D. W. (2000). Anatomy and discharge properties of pre-motor neurons in the goldfish medulla that have eye-position signals during fixations. *J. Neurophysiol.* 84, 1035–1049.

Aksay, E., Olasagasti, I., Mensh, B. D., Baker, R., Goldman, M. S., and Tank, D. W. (2007). Functional dissection of circuitry in a neural integrator. *Nat. Neurosci.* 10, 494–504. doi: 10.1038/nn1877

Arrenberg, A. B., Del Bene, F., and Baier, H. (2009). Optical control of zebrafish behavior with halorhodopsin. *Proc. Natl. Acad. Sci. U.S.A.* 106, 17968–17973. doi: 10.1073/pnas.0906252106

Debowy, O., and Baker, R. (2011). Encoding of eye position in the goldfish horizontal oculomotor neural integrator. *J. Neurophysiol.* 105, 896–909. doi: 10.1152/jn.00313.2010

Machens, C. K., and Brody, C. D. (2008). Design of continuous attractor networks with monotonic tuning using a symmetry principle. *Neural Comput.* 20, 452–485. doi: 10.1162/neco.2007.07-06-297

Pastor, A. M., Torres, B., Delgado-Garcia, J. M., and Baker, R. (1991). Discharge characteristics of medial rectus and abducens motoneurons in the goldfish. *J. Neurophysiol.* 66, 2125–2140.

Seung, H. S. (1996). How the brain keeps the eyes still. *Proc. Natl. Acad. Sci. U.S.A.* 93, 13339–13344. doi: 10.1073/pnas.93.23.13339
