# Supplementary material for: Optogenetic perturbations reveal the dynamics of an oculomotor integrator
Source: Front Neural Circuits. 2014 Feb 28;8:10. doi: 10.3389/fncir.2014.00010 (PMC3937552; doi:10.3389/fncir.2014.00010)
Supplement: Supplementary file 2 [file DataSheet1.ZIP › online_supp_mat/supp_mat_info.pdf]

Optogenetic perturbations reveal the dynamics of an oculomotor integrator

Online supplementary materials

Pedro J. Gonçalves, Aristides B. Arrenberg, Bastian Hablitzel, Herwig Baier, Christian K. Machens

MATLAB package for vector fields and Δθ vs. θ plots

A MATLAB package is provided, where the ILA model and the NP model are implemented and optogenetic stimulations can be performed on the left half of the integrator. The script has two options: **(a)** it produces a vector field where the user can observe the dynamics in an interactive way, and **(b)** it simulates the perturbation experiments, and generates Δθ versus θ plots. After unzipping the file zebrafish_integrator.zip, two files can be found:

• zebrafish_integrator_start.m: initialization program. All parameters used can be found in this file, within the function *pv_plot*.
• zebrafish_integrator_start.fig: graphical interface (not to open).

Below is a brief tutorial of the package.

1. Within the folder *zebrafish_integrator*, launch the program by entering *zebrafish_integrator_start* in MATLAB command line. A graphical interface appears.

2. In the panel *Model*, choose the model to be explored by clicking on the respective radio button. There are two options available: the *ILA model*, and the *NP model*.

3. In the panel *Type of stimulation*, choose the desired stimulation within the two options, *NpHR* (halorhodopsin) and *ChR2* (channelrhodopsin).

4. In the panel *Experiment*, two options are available: *Δθ/θ*, and *Interactive vector field*. *Δθ/θ* option generates Δθ versus θ plots similar to the ones provided in the paper. The plots correspond to averages over the number of iterations defined by the user (1 to 100 iterations), with white noise in the simulations and jittering in the intensities of stimulation. To run this option, select the respective radio button, choose the number of iterations on the slider just below, and click on the respective button *Run*. A progress bar appears and shows the estimated time left to generate the plot.

5. *Interactive vector field* produces the model dynamics in vector field format. To run this option, choose the respective radio button and click on the *Run* button just below, which

automatically plots the vector field in control conditions. Subsequently, the user can perform two actions:

• Stimulate the system with an amplitude defined by the slider *Stimulation amplitude*. The vector field is automatically updated, given the stimulation amplitude.

• Initialize the system within the vector field and observe its subsequent dynamics for 1.5 seconds, by clicking on the button *Trajectory* and selecting with the mouse the initial point in the vector field: a black dot corresponding to the system state subsequently follows a trajectory, which is plotted along the way. At the same time, the light stimulation, corresponding eye positions and movements are illustrated in the zebrafish animation on the right of the vector field. Note that although the displayed vector field corresponds to the stimulated case, the stimulation only occurs in the beginning of the trajectory (200 ms for NpHR and 100 ms for ChR2), the rest being governed by control case dynamics. Finally, we deliberately decelerated the dynamics during stimulation for the user to better follow the dynamics during this period.

Supplementary Movie 1

Supplementary Movie 1. NpHR stimulation after spontaneous saccades induces transient eye drifts.
Top: The eye movements of a *nacre-/-* mutant larva carrying the *Tg(UAS:NpHR-mCherry)s1989t* and *Et(E1b:Gal4)s1101t* transgenes were recorded under infrared light. The left hindbrain was stimulated during spontaneous eye movements. The time stamp (in seconds, first row), the stimulation magnitude of the laser (in volt, 4th row) and the angular eye positions (in degrees, 5th and 6th row) are plotted in the movie. Bottom: The eye positions of the left (red) and right (green) eye are plotted versus time. Each time the fish is stimulated (black trace) during left side eye positions (below the midline), the eyes transiently drift back.
